# Allosteric modulation of dopamine D_2L_ receptor in complex with G_i1_ and G_i2_ proteins: the effect of subtle structural and stereochemical ligand modifications

**DOI:** 10.1007/s43440-021-00352-x

**Published:** 2022-01-22

**Authors:** Justyna Żuk, Damian Bartuzi, Andrea G. Silva, Monika Pitucha, Oliwia Koszła, Tomasz M. Wróbel, Dariusz Matosiuk, Marián Castro, Agnieszka A. Kaczor

**Affiliations:** 1 Department of Synthesis and Chemical Technology of Pharmaceutical Substances with Computer Modeling Laboratory, Faculty of Pharmacy, Medical University of Lublin, 4A Chodźki St., 20093 Lublin, Poland; 2 Department of Pharmacology, Center for Research in Molecular Medicine and Chronic Diseases (CIMUS), Universidade de Santiago de Compostela, Avda de Barcelona, 15782 Santiago de Compostela, Spain; 3 Independent Radiopharmacy Unit, Faculty of Pharmacy, Medical University of Lublin, 4A Chodźki St., 20093 Lublin, Poland; 4 School of Pharmacy, University of Eastern Finland, Yliopistonranta 1, P.O. Box 1627, 70211 Kuopio, Finland

**Keywords:** Dopamine D_2_ receptor, GPCRs, Molecular dynamics, Molecular switches, Negative allosteric modulators, Positive allosteric modulators

## Abstract

**Background:**

Allosteric modulation of G protein-coupled receptors (GPCRs) is nowadays one of the hot topics in drug discovery. In particular, allosteric modulators of D_2_ receptor have been proposed as potential modern therapeutics to treat schizophrenia and Parkinson’s disease.

**Methods:**

To address some subtle structural and stereochemical aspects of allosteric modulation of D_2_ receptor, we performed extensive in silico studies of both enantiomers of two compounds (compound **1** and compound **2**), and one of them (compound **2**) was synthesized as a racemate in-house and studied in vitro.

**Results:**

Our molecular dynamics simulations confirmed literature reports that the R enantiomer of compound **1** is a positive allosteric modulator of the D_2L_ receptor, while its S enantiomer is a negative allosteric modulator. Moreover, based on the principal component analysis (PCA), we hypothesized that both enantiomers of compound **2** behave as silent allosteric modulators, in line with our in vitro studies. PCA calculations suggest that the most pronounced modulator-induced receptor rearrangements occur at the transmembrane helix 7 (TM7). In particular, TM7 bending at the conserved P7.50 and G7.42 was observed. The latter resides next to the Y7.43, which is a significant part of the orthosteric binding site. Moreover, the W7.40 conformation seems to be affected by the presence of the positive allosteric modulator.

**Conclusions:**

Our work reveals that allosteric modulation of the D_2L_ receptor can be affected by subtle ligand modifications. A change in configuration of a chiral carbon and/or minor structural modulator modifications are solely responsible for the functional outcome of the allosteric modulator.

**Graphical abstract:**

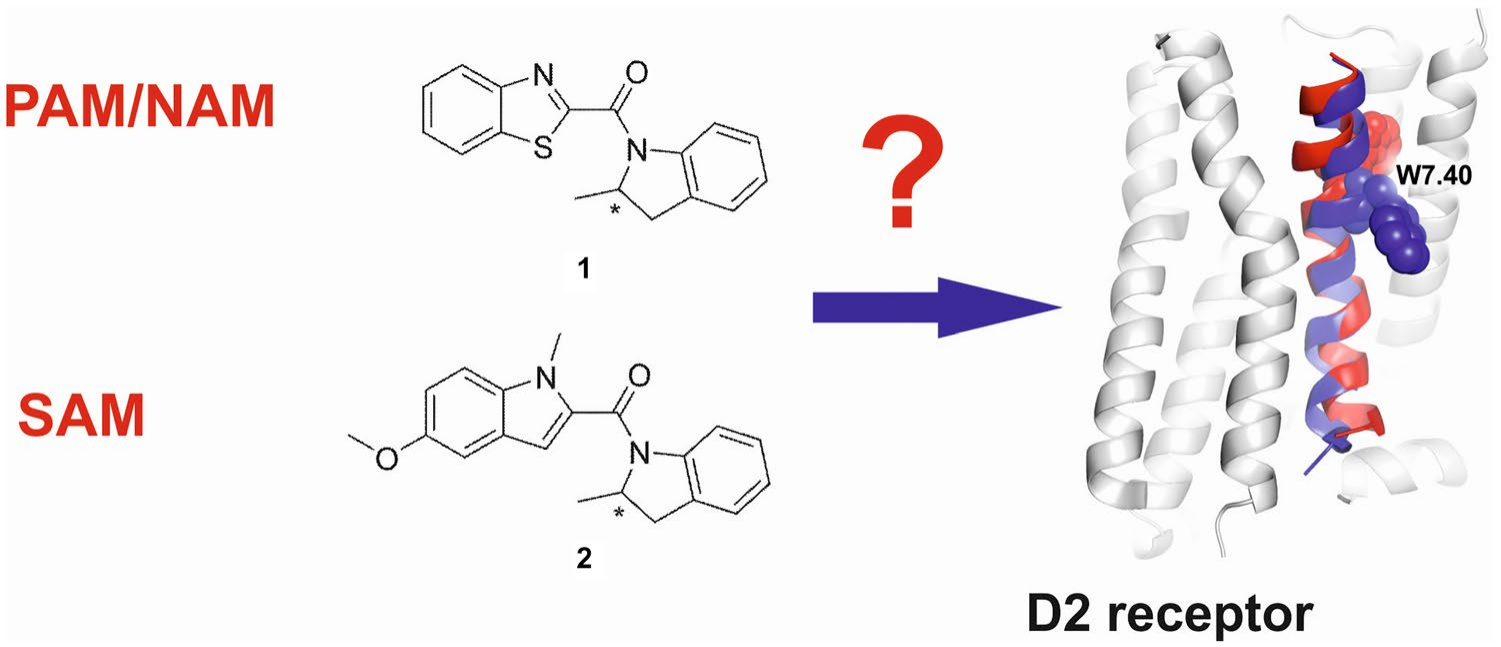

**Supplementary Information:**

The online version contains supplementary material available at 10.1007/s43440-021-00352-x.

## Introduction

Dopamine receptors belong to rhodopsin-like G protein-coupled receptors (GPCRs) and share the molecular architecture typical for this family of proteins. The gene for the dopamine D_2_ receptor (D2R) produces two isoforms: the truncated D_2SHORT_ receptor (D_2S_, UNIPROT accession code: P14416-2, 415 amino acids in length) and the D_2LONG_ receptor (D_2L_, UNIPROT accession code: P14416-1, 444 amino acids in length), containing an additional sequence of a 29-amino-acid fragment in intracellular loop 3, ICL3 [1]. While presynaptic D_2S_ receptors serve as auto-receptors in dopaminergic neurons inhibiting neurotransmission, D_2L_ receptors are mainly postsynaptic [2]. Still, both isoforms are co-expressed in the D2R-expressing neurons, share some pharmacological features [3–5] and support relevant postsynaptic dopamine functions [6].

A promising way of targeting GPCRs and achieving therapeutic effects with diminished risk of side effects is the use of allosteric modulators, which are compounds that interact with binding sites that are topographically different from the orthosteric site recognized by a native, endogenous ligand [7, 8]. The use of allosteric modulators has advantages over classical orthosteric modulators, among them the increased selectivity for GPCR subtypes, the so-called ‘ceiling effect’ that prevents overdosing, and the allosteric probe dependence, which offers the possibility of introducing beneficial therapeutic effects without compromising the integrity of complex, physiologically regulated signalling networks. Since allosteric modulators are very sensitive to protein conformational changes, they also have been used to determine whether a particular mutation produces global changes in protein conformation [9]. Allosteric ligands can be classified into three types depending on their pharmacological action. Positive allosteric modulators (PAMs) can potentiate agonist-mediated receptor responses, while negative allosteric modulators (NAMs) decrease receptor activity. Silent allosteric modulators (SAMs) can target allosteric binding sites and block positive or negative modulator activity rather than modulate orthosteric ligand responses [10–14]. At dopamine receptors, all three modes of allosteric modulation are possible [15].

A handful of ligands have been shown to modulate D2R by an allosteric mechanism. For example, the binding of sodium ions to D2R reduces its affinity for agonists by inducing conformational changes [16]. In contrast, the tripeptide proline-leucine-glycine (PLG) and a peptidomimetic, PAOPA, modify dopaminergic neurotransmission by increasing dopamine binding to D2R and prevent the conversion of high-affinity state to their low-affinity state [17–20].

**Fig. 1:**
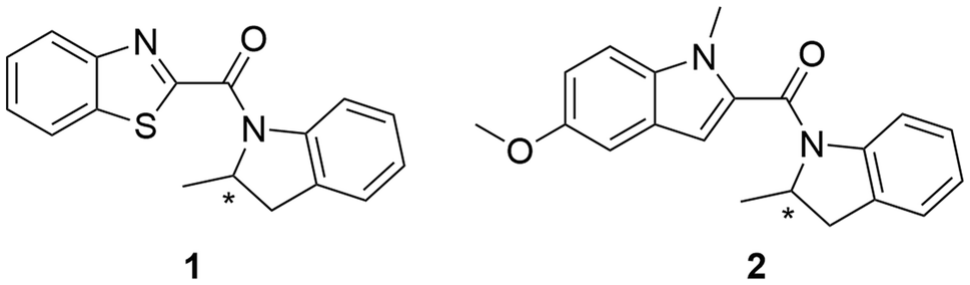
Structural formulas of the studied compounds **1** (21) and **2**

In this study, we present an extensive in silico and in vitro investigation of allosteric modulation of the D2R_LONG_ in complex with Gα_i1_ and Gα_i2_ proteins (DG1 and DG2) by compound **1** [21] and its novel derivative, compound **2** (Fig. 1). Wood et al. found that the R enantiomer of compound **1** does not directly stimulate the D2R, but potentiated the effects of dopamine. On the contrary, the S enantiomer reduced the effects of the PAM and the effects of dopamine. Finally, in radioligand binding studies, both enantiomers of compound **1** did not compete for the binding of orthosteric ligands. However, the R enantiomer resulted in a higher number of high-affinity sites for [^3^H]-dopamine, but did not affect *K*_d_. Compound **2** was designed and synthesized by our group, and it is reported here for the first time.

In this work, enantiomers of compounds **1** and **2** were studied, and their effects on the receptor function were investigated. The rationale for our work is constituted by the limited data on structural aspects of D2R allosteric modulation, in particular regarding the structure–activity relationship of the modulators.

## Materials and methods

### Chemistry

All reagents used for the synthesis were purchased from commercial suppliers and were used without further purification. NMR spectra were recorded on a Bruker AVANCE III 600 MHz spectrometer equipped with a BBO Z-gradient probe. Spectra were recorded at 25 °C using DMSO-d_6_ as a solvent with a non-spinning sample in 5 mm NMR-tubes. Chemical shifts were expressed in parts per million (ppm) using the solvent signal or TMS as an internal standard. High-resolution mass spectra (HRMS) were acquired on a Bruker microTOF-Q II mass spectrometer with electrospray ionization (ESI). Data were processed using MestReNova v.14.0.0 and Compass Data Analysis software. Spectra of the reported compound can be found in Supplementary Information.

#### Synthesis of 5-methoxy-1-methyl-1*H*-indol-2-yl)(2-methylindolin-1-yl)methanone (2)

A solution of ( ±)-2-methylindoline (0.243 mmol) in dry 1,2 dichloroethane (5 mL) was added to a solution of 5-methoxy-1-methyl-1*H*-indole-2-carboxylic acid (0.243 mmol) and *N*-(3-dimethylaminopropyl)-*N′*-ethylcarbodiimide (0.364 mmol) in dry 1,2-dichloroethane (15 ml). The reaction mixture was stirred for 24 h at room temperature, extracted with 3 N hydrochloric acid (2 × 2.5 ml), washed with water (2 × 1 ml) and dried with Na_2_SO_4_ for 24 h. The organic layer was distilled in vacuo and the residue was recrystallized from isopropanol. Yield: 51.2%. The product was obtained as a racemate and this form was used for subsequent studies.

^1^H NMR (600 MHz, DMSO-*d*_*6*_) δ 7.45 (d, J = 8.9 Hz, 1H), 7.31 (d, J = 7.3 Hz, 1H), 7.16–7.10 (m, 2H), 7.08–7.04 (m, 1H), 6.94 (dd, J = 8.9, 2.4 Hz, 1H), 6.82 (s, 1H), 4.89 (ddt, J = 10.8, 6.5, 3.2 Hz, 1H), 3.78 (s, 3H), 3.73 (s, 3H), 3.46 (dd, J = 16.0, 8.9 Hz, 1H), 2.68 (d, J = 15.9 Hz, 2H), 1.12 (d, J = 6.5 Hz, 3H).

^13^C NMR (151 MHz, DMSO-*d*_*6*_) δ 161.1, 154.0, 141.1, 133.7, 132.5, 131.5, 127.0, 126.3, 125.5, 124.0, 116.1, 113.9, 111.3, 102.2, 102.2, 56.8, 55.2, 35.3, 30.8, 21.2. HRMS (M + H) + calc. = 321.1598, exp. = 321.1591.

### Molecular modelling

Prior to molecular docking of allosteric ligands, the systems of D_2L_ receptor (with ICL3) in complex with the respective G protein immersed in the asymmetric membrane were built. The membrane environment was prepared using the CHARMM-GUI Membrane Builder server [22] and contains cholesterol, sphingomyelin, DOPC, DOPS, PLPC, POPC, POPE, POPG (proportions appropriate for membrane rafts [23]) and aqueous phase: TIP3P water molecules with 0.15 M NaCl. The study involved homology modelling of D_2L_ receptor with ICL3 in complex with Gα_i1_ or Gα_i2_, and 1 µs all-atom MD simulations of the systems before docking allosteric ligands has been described in detail in a previous paper [24]. The recent crystallization of the dopamine D2R in the active conformation [25] (PDB ID: 6VMS) provides detailed insights into the receptor structure and its activation mechanisms. However, this work is based on a complete dopamine D2R by homology modelling and submitted to 1 µs MD simulations. To compare the crystal form of the D_2_ receptor with our model, we calculated RMSD for Cα atoms of eight helices: 1.74 Å for DG1 (D2LONG receptor in complex with G_i1_ protein) and 2.29 Å for DG2 (D2LONG receptor in complex with G_i2_ protein).

The structures of allosteric ligands were modelled using the Hartree–Fock approach and 6-31G* basis set of Spartan v. 10 VI.0.1 [26]. The hypothetical allosteric binding pockets for the studied ligands were determined by docking performed by Molegro Virtual Docker 6.0 software [27] using the following settings: number of runs = 100; the maximal number of iterations = 10,000; the maximal number of poses = 50; and the poses representing the lowest value of the scoring function (MolDockScore) were further analysed. Molecular dynamics simulations were done in Gromacs v. 2018.4 [28]. An Amber03 force field [29] was used for receptors, Slipids (Stockholm lipids) [30] for the membrane and General Amber Force Field (GAFF) [31] for ligands. Ligand ESP charges were obtained by RESP ESP charge Derive Server [32]. Topologies were obtained with the ACPYPE server [33]. Each system was minimized for 500 steps and equilibrated in 1 ns NVT and 10 ns NPT simulations with protein and ligand position restrained by a force constant of 10,000 kJ/mol nm^2^ put on the heavy atoms. The most energetically favourable orientations (one for each system with lower protein − ligand interaction energy) were subjected to 1 µs all-atom molecular dynamics run in triplicate. As a reference, these systems without modulator (with dopamine) were also simulated. Standard Gromacs tools, VMD v. 1.9.3 [34], PyMol v. 4.6 [35] and Maestro Schrödinger v. 12.4 software [36] were used for data extraction and analysis of the results. In particular, gmx covar and gmx anaeig were used for principal component analysis. For the first analysis, all trajectories were concatenated and analysed in a common subspace. For further analyses, trajectories containing G_i1_ and G_i2_ proteins were grouped separately to avoid G protein-dependent bias. Trajectories were fitted to Cα of the 7TM bundle (without ICL3). Analyses were performed on heavy atoms of separate helices.

The similarity analysis was performed using Canvas v. 4.2 [37, 38]. The structures of compounds **1** and **2** were compared to the structures of 10,054 dopamine D_2_ receptor ligands with K_i_ below 10,000 nM as available in the CHEMBL database [39]. Hashed linear fingerprints and Tanimoto similarity were used.

The molecular similarity approach as incorporated in PASS software [40] was applied to identify other possible pharmacological activities, biological targets and adverse effects of compounds **1** and **2**.

### In vitro studies

#### Competition radioligand binding assays at D_2_ receptors

D_2_ receptor binding assays were performed in membranes from CHO-K1 cells stably expressing the cloned human D_2S_ receptor previously described [41]. Competition binding experiments were carried out following previously described procedures [42]. In brief, cell membranes and 1.5 nM radioligand [^3^H]-Spiperone (76.1 Ci/mmol, 1 mCi/ml, NET1187250UC, PerkinElmer, Madrid, Spain) were incubated in 96-well assay plates for 120 min at 25 °C in incubation buffer (50 mM Tris–HCl, 120 mM NaCl, 5 mM KCl, 5 mM MgCl_2_, 1 mM EDTA (pH = 7.4)), in the absence or presence of compound or vehicle (dimethyl sulfoxide (DMSO)). After incubation time, assay plates were filtered through GF/C glass filters and filters washed with ice-cold wash buffer (50 mM Tris–HCl, 0.9% NaCl (pH = 7.4)). Nonspecific binding was assessed in wells containing 10 µM sulpiride ((S)-( −)-Sulpiride, Sigma-Aldrich). Compound **2** was assayed at concentrations from 1 nM to 10 µM. The compound was dispensed into the empty assay plate using an acoustic dispensing noncontact instrument and vehicle (0.1% DMSO) was kept constant along the concentration curve. Nephelometry confirmed the solubility of the compound at these concentrations in the assay buffer and assay conditions, whereas 100 µM concentration was excluded from the assays due to solubility problems as assessed by nephelometry (NEPHELOstar Plus instrument, BMG LABTECH GmbH, Ortenberg, Germany), see Fig. S14. Haloperidol (Sigma-Aldrich) (0.01 nM–1 µM) was included as reference competitor in the experiments.

#### cAMP assays at D_2_ receptors

Direct D_2_ agonist or antagonist activity of compound **2** was investigated in cAMP assays on the cell line stably expressing the cloned human D_2S_ employed in the radioligand binding assays. Previously described protocols were followed with minor modifications [42]. Cells were seeded in 384-well plates in assay buffer containing 500 µM 3-isobutyl-1-methylxanthine (IBMX) (as inhibitor of cAMP-specific phosphodiesterases, directly added as powder to the assay buffer). Vehicle (1% DMSO) or antagonist (10 µM compound **2**) was added to the corresponding wells (“antagonist mode”) by traditional tip-based dispensing method. After 5 min incubation at 37 °C, 10 µM compound **2** or quinpirole (( −)-Quinpirole hydrochloride, Sigma-Aldrich) at the concentration of 100 nM close to its EC_50_ and prepared from aqueous stock solution, were added as agonists to the corresponding wells (“agonist mode”). After 10 min incubation at 37 °C, 10 µM forskolin (from a 5x intermediate solution prepared in assay buffer containing IBMX and 0.5% DMSO) was added to the corresponding wells (“agonist mode” and “antagonist mode”) and incubation was continued for 5 min. After this time, cellular cAMP levels were quantified using the homogeneous time-resolved fluorescence (HTRF)-based cAMP Gs dynamic kit (Cisbio, Bioassays, Codolet, France) according to the manufacturer’s protocol. Basal cAMP levels were determined in wells in the absence of forskolin, both in the presence of vehicle and compound **2**.

Potential properties of compound **2** as allosteric modulator of D_2_ receptors were assessed by investigating its effect on dopamine response at cAMP signalling. Concentration (1 nM–100 µM)–response curves of dopamine (Dopamine hydrochloride, Sigma-Aldrich) (prepared from aqueous stock solution) were carried out in the presence of vehicle (1% DMSO) or 10 µM compound **2** on the cell line stably expressing D_2_ receptors indicated above. Cells were incubated with the ligands and 10 µM forskolin for 1 h at room temperature according to protocols previously described [21], and cellular cAMP levels were determined as indicated above. Basal cAMP levels were determined in wells in the absence of dopamine and forskolin, both in the presence of vehicle and compound **2**. Individual concentration–response curves were fitted to the model of sigmoidal dose–response curve log(agonist) vs. response (three parameters) (Hill slope (n_H_) = 1) described by the equation Y = bottom + (top–bottom)/(1 + 10^((LogEC50-X))) using Prism 7 software (GraphPad, San Diego, CA) and pEC_50_ values for dopamine were extracted from the fitting. Dopamine response at concentrations EC_80_–EC_90_ and EC_30_ corresponds to the experimental data points at the concentration of dopamine that elicited the response closest to 80–90% (or to 30%) of the maximal dopamine response in concentration–response curves of dopamine in the absence of vehicle or compound **2**.

The solubility of compound **2** at the concentration employed in cAMP assays was confirmed by nephelometry, assessed as previously indicated (see Fig. S15).

#### Statistical analysis

Two-way ANOVA and Sidak's multiple comparisons test were employed for comparison of the effect of vehicle versus compound **2** (from 1 nM to 10 µM) in radioligand binding displacement curves. In functional assays of cAMP signalling, one-way ANOVA and Sidak's multiple comparisons test were employed for comparison of the effect of vehicle versus 10 µM compound **2** on basal and forskolin-stimulated conditions, whereas unpaired *t* test was employed for comparison of 100 nM quinpirole response on forskolin-stimulated cAMP production in the presence of vehicle versus 10 µM compound **2**, as well as for comparison of dopamine EC_30_ and EC_90_ responses on forskolin-stimulated cAMP production in the presence of vehicle versus 10 µM compound **2**, and for comparison of dopamine pEC_50_ values in the presence of vehicle versus 10 µM compound **2.**

## Results

### Chemistry

**Scheme 1:**
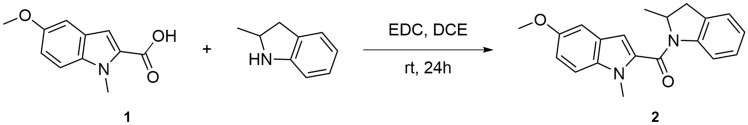
**1.** Synthesis of compound **2**. Reagents and conditions: EDC—*N*-(3-dimethylaminopropyl)-*N*′-ethylcarbodiimide, DCE—1,2-dichloroethane, rt—room temperature

Compound **2** was synthesized by a simple condensation of racemic 2-methylindoline with 5-methoxy-1-methyl-1*H*-indole-2-carboxylic acid using *N*-(3-dimethylaminopropyl)-*N*′- ethylcarbodiimide (EDC) as a coupling agent (Scheme 1). The reaction was conducted in 1,2-dichloroethane (DCE) at room temperature over 24 h. The product was obtained as a racemic mixture and used as such in subsequent investigations. The identity of compound **2** was confirmed by ^1^H NMR, ^13^C NMR and HRMS.

### In silico studies

To estimate the structural novelty of the studied compounds, their structures were compared with the structures of 10,054 dopamine D_2_ receptor ligands with K_i_ < 10,000 nM available in CHEMBL database. Canvas 2.4 software was used for Tanimoto similarity evaluation. Regarding compound **1**, three most similar D_2_ receptor ligands exhibit very low similarity of 0.247. Concerning compound **2**, one compound exhibited the highest similarity of 0.250. The summary of this data is shown in Table S1 in Supplementary Information.

To find other possible biological effects and other possible biological targets of the compounds, PASS software was used. PASS software relies on 2D comparison of the studied compound with the compounds of known activities in its database. In general, no significant results (probability that the compound is active, *Pa* > 0.7) were found. Compound **1** may exert nootropic properties (*P*_*a*_ = 0.573), while compound **2** could be 5-hydroxytryptamine release stimulant (*P*_*a*_ = 0.523). No anti-targets or toxic/adverse effects have been found for both compounds. The summary is presented in Table S2 in Supplementary Information. These results should be interpreted having in mind that the lack of identified significant additional activities may be connected with the structural originality of the studied compounds.

#### Identification of receptor binding sites

To date, no D_2_ receptor–allosteric modulator complex structure is known from X-ray or electron microscopy studies. Therefore, careful inspection of molecular docking results of the enantiomers of compounds **1** and **2** (R1, S1, R2 and S2) to the D_2_ receptor models coupled with Gα_i1_ or Gα_i2_ protein (R1_DG1, S1_DG1, R1_DG2 and so forth, see Table 1) was crucial for the identification of a putative allosteric binding pocket of the dopamine D_2_ receptor.

**Table 1:** The studied modulator–D_2_ receptor complexes

Compound	Enantiomer	Gα protein	Complex symbol
1	R	Gα_i1_	R1_DG1
1	R	Gα_i2_	R1_DG2
1	S	Gα_i1_	S1_DG1
1	S	Gα_i2_	S1_DG2
2	R	Gα_i1_	R2_DG1
2	R	Gα_i2_	R2_DG2
2	S	Gα_i1_	S2_DG1
2	S	Gα_i2_	S2_DG2

The docking area was defined at the extracellular part of the receptor above the dopamine-binding site. The resulting pose clusters were analysed. The most frequently occurring and best scored putative allosteric site was similar to that described in the literature [43–50], i.e. at the extracellular ends of TM2 and TM7. Therefore, this pocket was selected for further studies. As compounds **1** and **2** are close structural analogues, a similar binding site was assumed, which was supported by molecular docking. The most favourable orientation of R and S was with the benzothiazole group pointing to the extracellular side of the protein. The binding pocket of R1_DG1 consists of W2.60, V2.61, L2.64, V3.29, F6.51, H6.55, I6.59, Y7.34, T7.38 and Y7.42 (Ballesteros–Weinstein residue numbering [51]). The most convincing docking poses (i.e. corresponding to allosteric ligands poses known from the literature, selected by visual inspection, with high scoring values) involved the formation of hydrogen bonds between the benzothiazole nitrogen of the ligand and Y7.34. In S1_DG1, the putative binding pocket consists of V2.61, L2.64, D3.32, V5.40, F6.51, H6.55, Y7.34, T7.38 and Y7.42. The best-scoring orientation of R2 and S2 involved a 4-methoxy-1-methylindole substituent pointing to the extracellular side of the protein, close to TM2, TM6 and TM7. The binding regions consist of W2.60, V2.61, L2.64, E2.64, F6.51, H6.55, S7.35, Y7.34, T7.38 and Y7.42. In both R2_DG1 and S2_DG1, the *carbonyl oxygen* of the ligand shows interactions with the Y7.34. In DG2, the best-scoring orientations were located closer to the extracellular side of the receptor than in DG1. The best poses appeared in the proximity of TM2, TM6 and TM7 and were surrounded by L2.64, H6.55, N6.58, I6.59, P7.31, and Y7.34 for all the studied ligands. The hydrogen bonds were observed between the carbonyl oxygen of the ligands and N6.58.

#### R1 bound to DG1

**Fig. 2:**
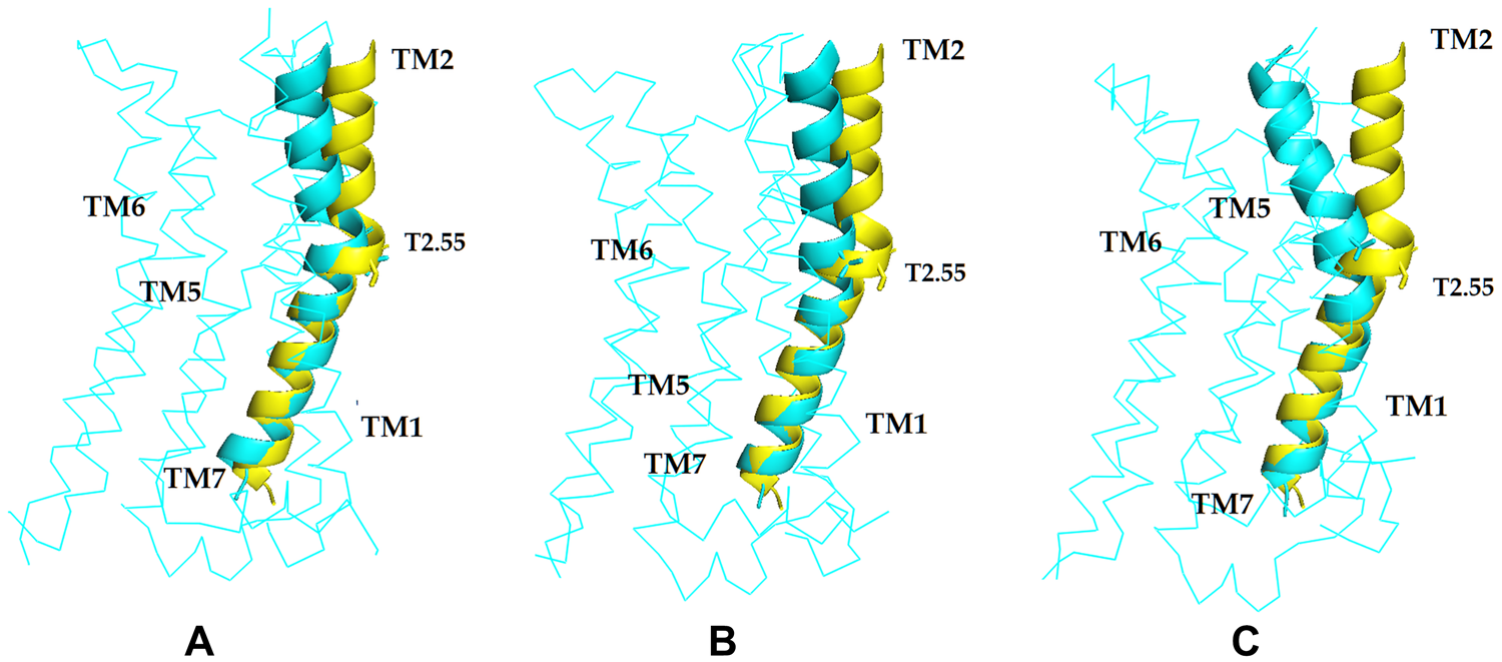
The superimposition of TM2 of dopamine_DG1 complex simulation (yellow) and TM2 of three replicas (**A–C**) of R1_DG1 (cyan) after 1 µs MD simulations with conformational state described above. The structures are shown as cartoon for TM2 and ribbon for the rest of the receptor. ICL3 was truncated for clarity

The RMSD values for each helix, calculated from molecular dynamics simulations for starting and final conformations, indicate increase in motility of TM2, TM6 and TM7 upon modulator binding (average for three replicas: 1.4 Å, 1.1 Å and 1.2 Å, respectively), compared to dynamics in absence of any modulator (0.8 Å, 0.8 Å and 0.9 Å, respectively). A significant change in TM2 conformation, involving inward helix bending above T2.55, was observed in all three replicas. Figure 2 shows the TM2 conformations after 1 µs MD simulations for three R1_DG1 replicas superimposed with the modulator-free complex. All the R1_DG1 simulations show significant deviation of the extracellular part of TM2 from the structure of the modulator-free receptor.

**Fig. 3:**
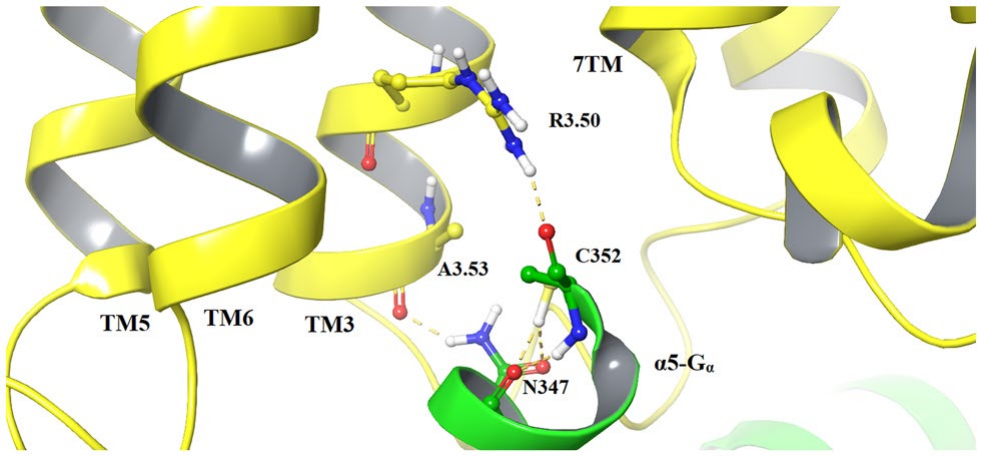
Crucial amino acid interactions within G protein coupling domains in R1_DG1 complexes. The structure of D2R is shown as yellow ribbons and the C-terminal part of α5-Gα as a green ribbon, whereas important amino acids are highlighted as sticks. Hydrogen bonds are marked as yellow dashes

Ligand-specific GPCR conformational changes involve receptor domains engaged in the G protein coupling. Our receptor model was built with a complete intracellular loop 3 (ICL3) [24], enabling analysis of the receptor–G protein interactions. The highly conserved DRY motif at the intracellular end of TM3 is known to be involved in the process via R3.50 [52, 53]. In all simulations, R3.50 formed an ionic interaction with C352 of the α5 helix of Gα (α5-Gα) (Fig. 3). This interaction remained stable in dopamine_DG1 complex MD simulation, similarly to the interaction between N347 of α5-Gα and A3.53.

#### R1 bound to DG2

Simulations of the modulator bound to D2R in complex with G_i2_ yielded similar results, with additional changes in the cytoplasmic and extracellular side of the TM region. More motility and rearrangements for R1_DG2 were observed in TM5, TM6 and TM7 (average for three replicas: 1.3 Å, 1.4 Å and 1.3 Å, respectively), with their intracellular segments undergoing an outward rigid body movement. RMSD values for dopamine_DG2 complex for TM5, TM6 and TM7 were 0.8 Å, 0.9 Å and 0.9 Å, respectively. The rotation of TM6 towards TM5 and slight bending at the W6.48 allowed a decrease in TM5–TM7 distance. At the end of the simulation, the Cα atoms of Y5.58 and Y7.53 are separated by < 10 Å, while in the simulation of dopamine-DG2, these tyrosines are separated by > 12 Å (not shown).

**Fig. 4:**
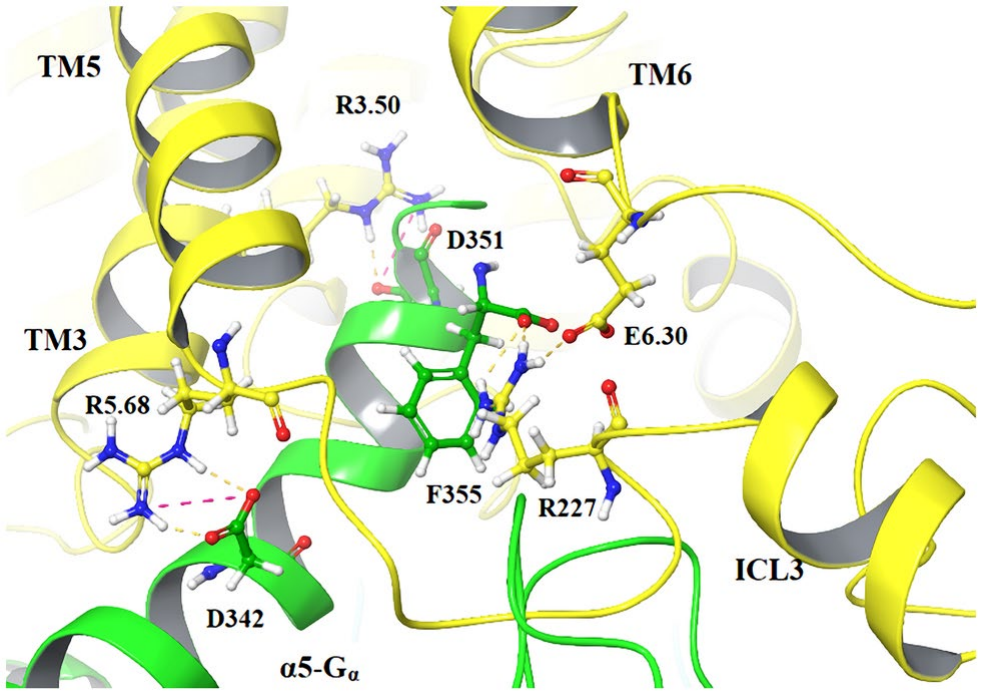
Crucial amino acid interactions within G protein coupling domains in R1_DG2 complexes. The structure of D2R is shown as yellow ribbons and C-terminal part of α5-Gα as a green ribbon, whereas important amino acids are highlighted as sticks. Hydrogen bond marked as yellow dashes and salt bridges as pink dashes

The deeper binding of the α5-Gα domain in the intracellular G protein-binding receptor cavity yielded more protein–protein interactions (Fig. 4). The C-terminus of Gα protein is surrounded by TM3, TM5 and TM6 of the receptor. The conformation of R227 (ICL3) is stabilized by interaction with the C-terminal part of TM6. In particular, the E6.30 side chain formed contacts with the F355 residue of the Gα protein. The position of Gα protein allowed R3.50 to form polar interaction with D351, and R5.68 residue interacts with D342. These receptor–G protein contacts were maintained throughout all three replicas. The dopamine_DG2 complex simulation shows only two stable hydrogen bonds: R3.50/D351 and K6.32/F355.

#### S1 bound to DG1 and DG2

The structures of S1_DG1 and S1_DG2 differ significantly from the receptor–dopamine and R1 modulator complexes. MD trajectories for S1_DG1 and S1_DG2 show a coordinated extracellular opening and intracellular closure of the protein. In both S1_DG1 and S1_DG2 complexes, RMSD for each helix showed increased values for TM5, TM6 and TM7 (average for three replicas: 1.7 Å, 1.5 Å, 1.5 Å in DG1, respectively and 1.1 Å, 1.9 Å, 1.3 Å in DG2, respectively). In all simulations, TM6 is seen to rotate towards TM7 orienting M6.36 into the central part of the receptor. Simultaneously, the distance between TM5 and TM6 increases, while the intracellular tail of TM7 bends outward, increasing its distance to TM5 (measured between Cα atoms of Y5.58 and Y7.53) and decreasing the distance to TM1.

**Fig. 5:**
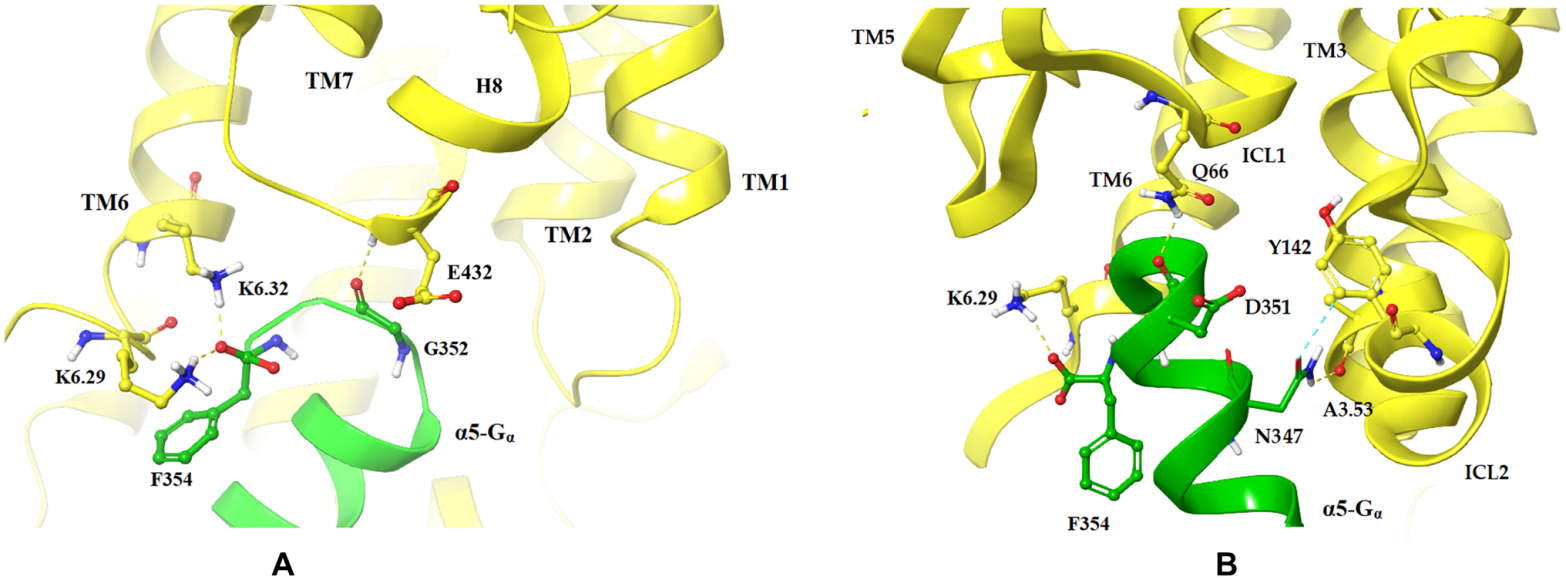
Crucial amino acid interactions within G protein coupling domains in S1_DG1 (**A**) and S1_DG2 (**B**) complexes. The structure of D2R is shown as yellow ribbons and C-terminal part of α5-Gα as a green ribbon, whereas important amino acids are highlighted as sticks. Hydrogen bonds are marked as yellow dashes, salt bridges as pink dashes and π interaction as blue dashes

Compared to dopamine-bound R1_DG1 and R1_DG2 complexes, in the S1 simulations, the α5-Gα–D2R interface is shaped differently. The binding of the C-terminus of Gα_i_ protein is much more shallow and involves different interactions of ICL1 (D351/Q66), TM3 (N347/A3.53), ICL2 (N347/Y142), TM6 (F354/K6.29) in S1_DG1 complex and TM3 (R3.50/D351) and TM6 (K6.32/F354) in S1_DG2 complex (Fig. 5).

#### R2 and S2 bound to DG1 and DG2

The RMSD values and the visual inspection of trajectories for both compound 2 enantiomers show that receptor fluctuations are comparable with modulator-free complexes. The differences in RMSD values between modulator-bound and modulator-free receptor complexes were below 0.2 Å. The conformational changes of helices are similar to modulator-free complexes, making R2 and S2 efficacy undetectable by MD.

**Fig. 6:**
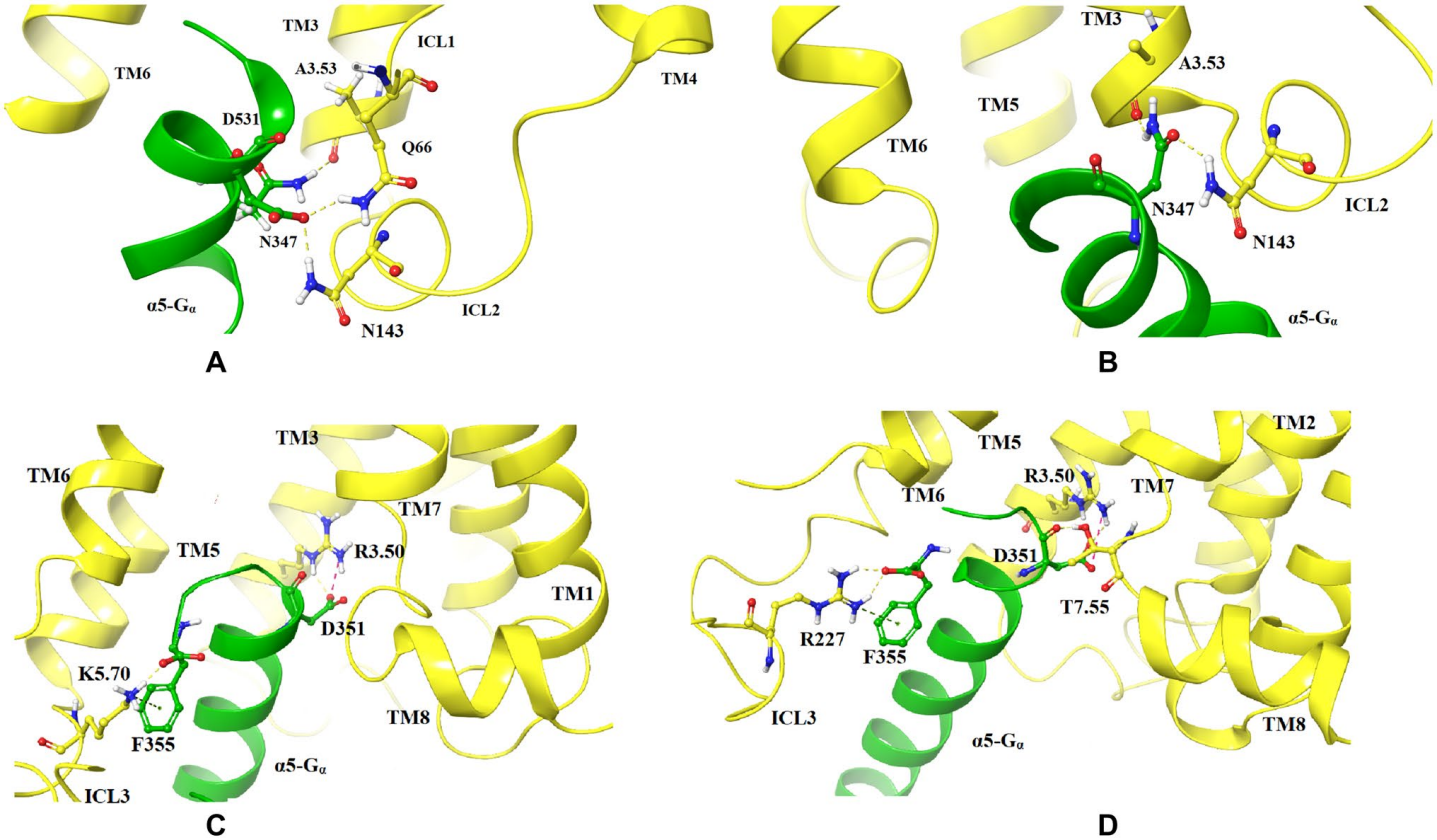
Crucial amino acid interactions within G protein coupling domains in R2_DG1 (**A**), S2_DG1 (**B**), R2_DG2 (**C**) and S2_DG2 (**D**) complexes. The structure of D2R is shown as yellow ribbons and C-terminal part of α5-Gα as a green ribbon, whereas important amino acids are highlighted as sticks. Hydrogen bonds are marked as yellow dashes and salt bridges as pink dashes

Figure 6 shows the last representative snapshots of R2 and S2 complexes, in which the effect of the modulators on the conformation of the α5-Gα-D_2_ receptor interface is compared. Compared to the simulations of the dopamine_DG1 complex, in R2_DG1 and S2_DG1, a similar arrangement of the α5-Gα domain in the receptor bundle can be observed. In the last frame of the simulation, a hydrogen bond connecting N347 of α5-Gα domain with A3.53 and two stable hydrogen bonds, N143 from ICL2 with D351 and Q66 from ICL1 with D351, are seen (A). In the S2_DG1 complex simulation, two hydrogen bonds N347/A3.53 and N347/N143 of ICL2 (B), were formed. In the case of the R2_DG2 simulation, the D351 residue of the C-terminus of Gα_i_ protein forms polar interaction with R3.50 of the DRY motif. The hydrogen bond and π–cation interaction between D351 and K5.70 are also visible (C). The simulation of the S2_DG2 complex shows stable hydrogen bonds R3.50/D351, T7.55/D351 and π–cation interaction R227/F355 involving ICL3 (D).

#### Molecular switches

Figures S1–S10 show the action of particular molecular switches in all the simulated complexes, including the time evolution of dihedral values. The most apparent difference between compounds is the Y5.58–Y7.53 distance. In positively modulated receptor (R1-containing complexes), the distance decreases in all simulations, regardless of G protein type. In the presence of the negative modulator (S1), the distance increases. Meanwhile, R2 and S2 compounds do not seem to affect it significantly, and the only observable difference is the fluctuation induced by R2, opposed to relatively stable values in S2 complexes.

Another apparent link between protein conformation and the bound modulator was found at the NPxxY motif and neighbouring F6.44 residue. In R1 complexes, Y7.53 prefers χ_1_ dihedral values of 100°, while in S1 complexes, it usually rotates to − 100°. Similarly, F6.44 seems to be affected, and assumes χ_1_ values of − 100 in R1_DG1 complexes and switches to ca. 100° in S1_DG2 complexes, while in G_i2_-coupled receptors it frequently fluctuates between both states. R2 and S2 simulations are generally characterized by increased motility of both aromatic residues.

#### Interactions of allosteric modulators with the receptor

**Fig. 7:**
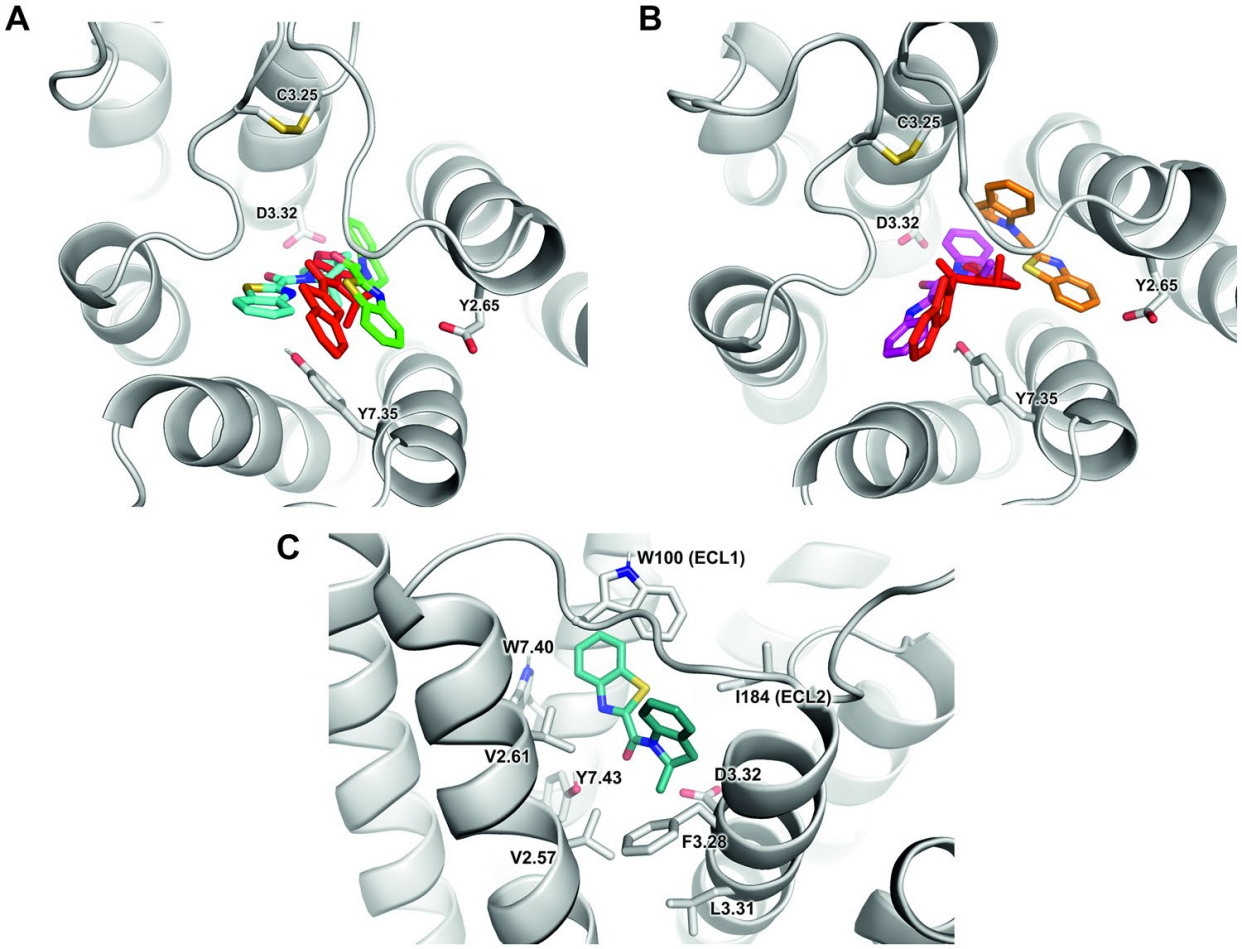
Representative poses of modulators (R1 and S1) after molecular dynamics simulations. **A** Drift of the S1 modulator from the initial docking position (red) through simulations. **B** Drift of the R1 modulator from the initial docking position (red) through simulations may result in a binding pose (green) distinct from that of S1. **C** The unique binding pose of R1 modulator shown from perspective of TM2, TM3 and ECL1

The representative poses of R1 and S1 modulators in complex with D_2_ receptor after molecular dynamics simulations and their comparison with the initial docking poses are shown in Fig. 7. To improve clarity, the G_i1_-bound receptor was used as an example. The (S) enantiomer of compound **1,** which is NAM, does not significantly drift from the initial docking pose, which is shown in Fig. 7A. In turn, in simulations of its (R) enantiomer, both maintaining the initial pose and drift to other poses were seen, suggesting that the initial docking pose was not optimal (Fig. 7B). The representative conformation of R1 modulator after drift from the initial pose is shown in Fig. 7C. The pose is particularly interesting, as its analysis sheds some light on hypothetical mechanisms of its positive modulatory effects, as well as provides hypothetical explanation of different signalling outcomes of the two enantiomers of compound **1**. In this pose, the methyl group at the chiral carbon protrudes into a hydrophobic pocket formed by F3.28, L3.31 and V2.57, resulting in favourable shape complementarity and removal of water from the hydrophobic area. In this particular conformation, the R1 modulator does not prohibit entrance of small molecule ligands into the orthosteric pocket. Moreover, its carbonyl group would serve as additional coordination of positively charged protonated nitrogen of orthosteric ligands. Binding of the (S) enantiomer in an analogical way would place the methyl group in immediate proximity of the aspartate 3.32, making the modulator binding much less energetically favourable. The binding of R2 and S2 compounds would also be less favourable, since N-methylation would affect possible direct or water-bridged polar interactions with Y7.43. Meanwhile, the (S) enantiomer of compound **1** prefers to remain in the position that hinders the entrance of any ligands to the orthosteric binding pocket. On the basis of the presented final complexes, further strategies of ligand modifications could be proposed. Given the complementarity of the indoline moiety with the area under the ECL1, the modification attempts should be focused on the benzothiazole part, avoiding bulky substituents that could interfere with π–π stacking between the moiety and surrounding tryptophan residues (W100 and W7.40).

To better understand the action of the studied modulators at the dopamine D_2_ receptor, the distance matrices were calculated with Gromacs tools (Supplementary Information, Figs. S9A and S10A). The last 200 ns of simulations were considered. On the distance maps, points corresponding to values of 0–5 Å are marked as black dots, and distances between modulators and D_2_ receptor residues are surrounded by circles. The detailed analysis of contact maps can be found in the Supplementary Information.

#### Principal component analysis

**Fig. 8:**
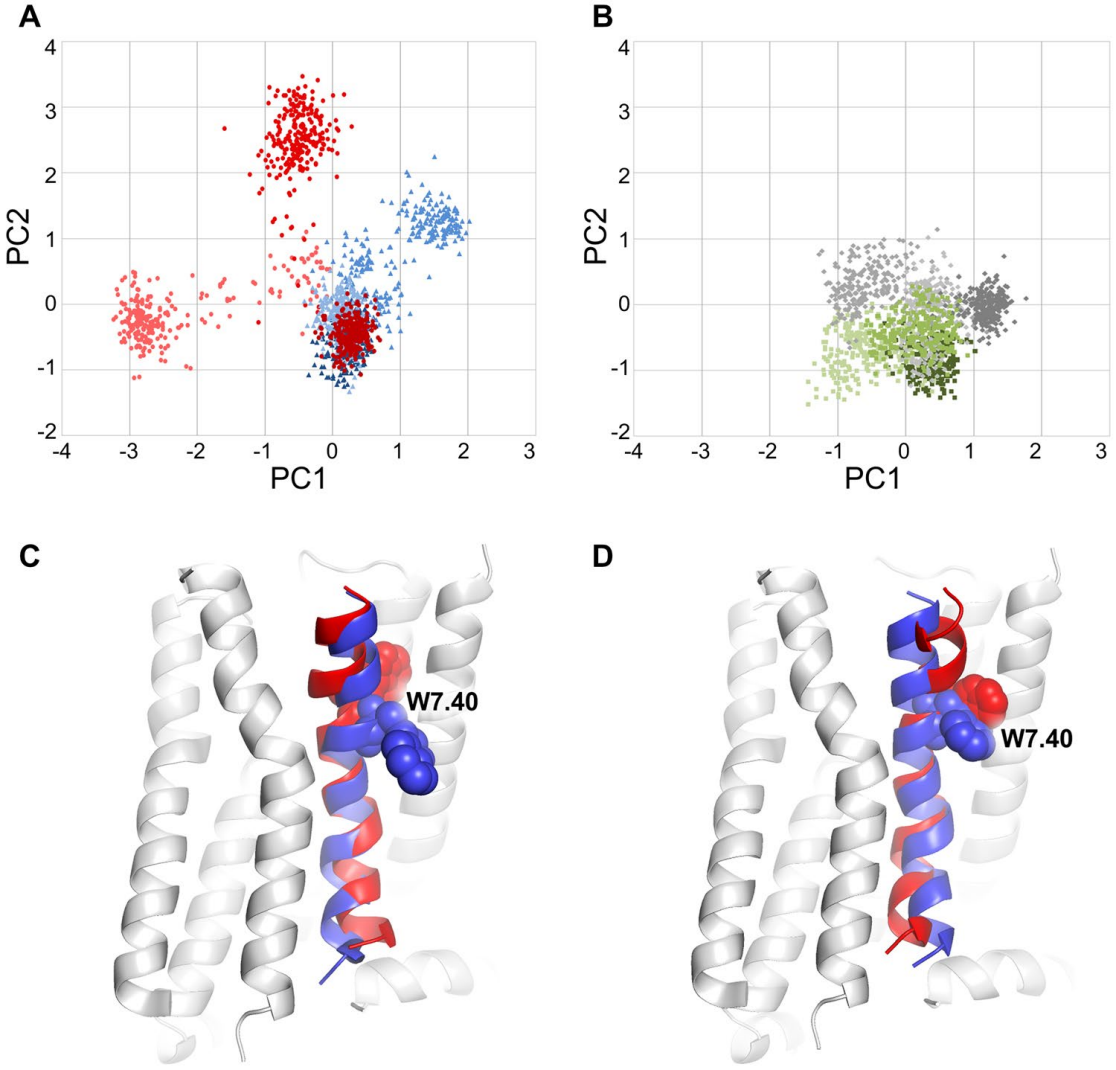
Statistical analysis of relationship between modulator type and motions of TM7 in G_i1_-bound complexes. **A** and **B** Conformational space explored by enantiomers of compound 1 and 2, respectively, in terms of PC1 and PC2. Analysis was performed in a common space, and values presented in shades of red represent simulations with R enantiomer of compound 1 and simulations of the S enantiomer presented in blue. Conformations induced by the R enantiomer of compound 2 are presented in green, while tose of the S enantiomer in gray. **C** and **D** Projections of extreme PC values on trajectories of TM7 in terms of PC1 and PC2, respectively, with a model of the receptor in the background for the context. Colour coding of TM7 conformations corresponds to panel A

**Fig. 9:**
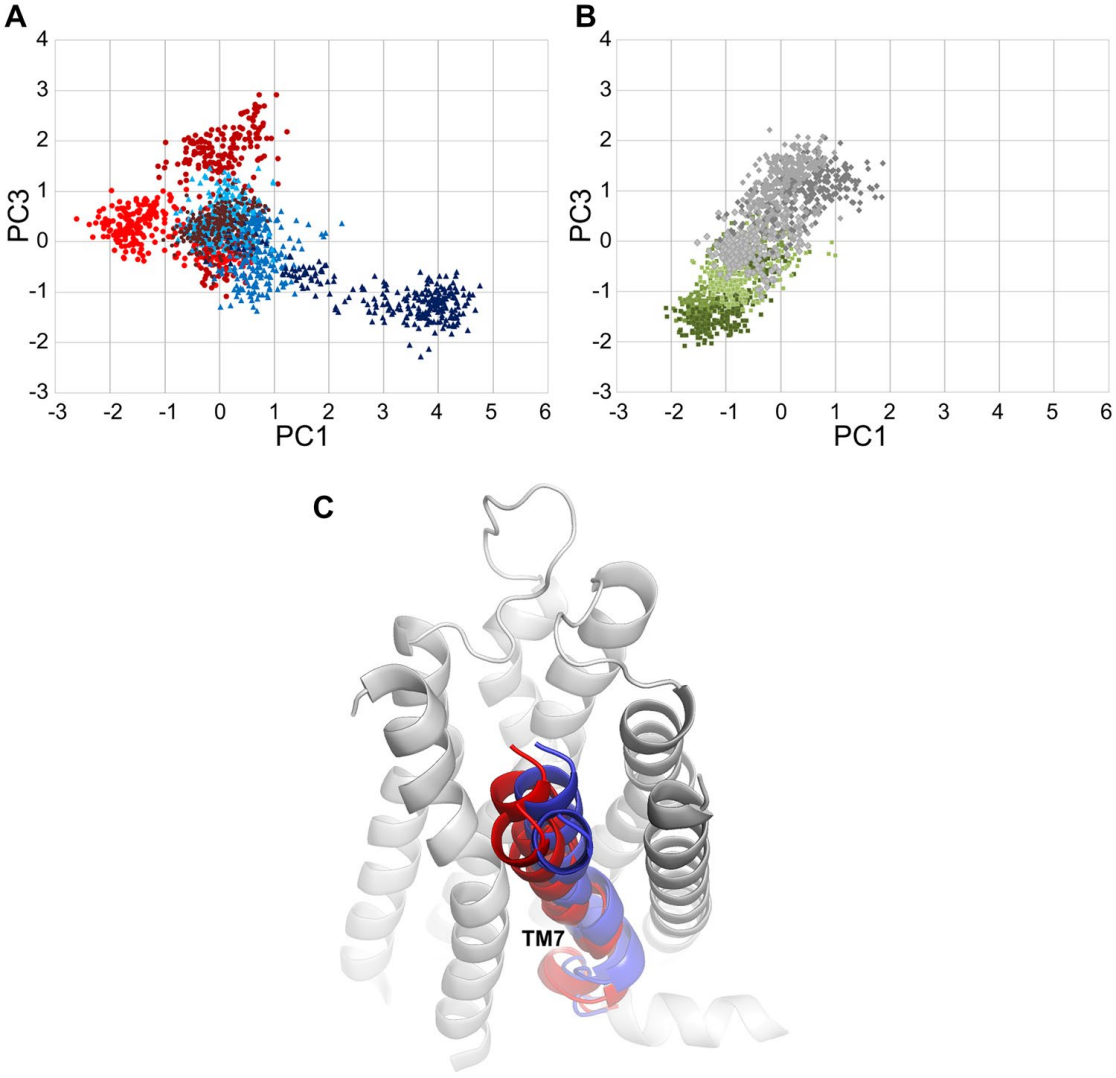
Statistical analysis of relationship between modulator type and motions of TM7 in G_i2_-bound complexes. **A** and **B** Conformational space explored by enantiomers of compound 1 and 2, respectively, in terms of PC1 and PC3. Shades of red represent simulations with R enantiomer of compound 1, and simulations of the S enantiomer presented in blue. Conformations induced by the R enantiomer of compound 2 are presented in green, while those of the S enantiomer in gray. Trajectories containing PAM are grouped in the upper left part of the diagram, while NAM-containing systems are apparent in the lower right. Simulations with SAM are grouped along a diagonal separating simulations with PAM and SAM. **C** Projections of extreme PC values on trajectories of TM7 in terms of PC1 and PC2, overlapped in one frame, with a model of the receptor in the background for the context. Colour coding of TM7 conformations corresponds to panel A. Decreased distance to TM6 is a common feature of low PC1 values and high PC3 values, corresponding to space occupied by PAM-containing complexes. Analogically, high PC1 values and low PC3 values are characterized by decreased distance between TM7 and TM2

**Fig. 10:**
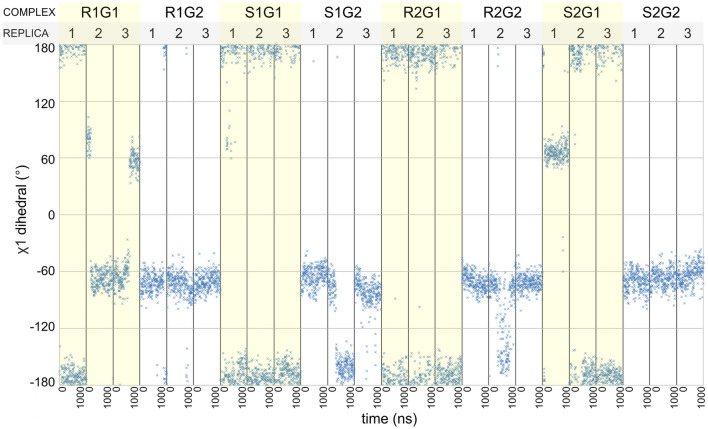
Values of χ_1_ dihedral of W7.40 in all simulations

To find statistically relevant relationships between modulator structure and the protein conformation, as well as for additional validation of the SAM mechanism of enantiomers of compound **2**, principal component analysis (PCA) was employed (Figs. 8, 9 and 10).

PCA performed for the whole 7TM bundle (with ICL3 truncated) revealed significant differences between the receptor conformations in complex with G_i1_ and G_i2_ proteins (Fig. S11). For this reason, to avoid domination of G protein subtype-dependent differences in the results, all further PCAs were done separately for G_i1_ and G_i2_ bound receptors.

The most pronounced differences between positive, negative and silent modulators were found in conformation of TM7. The first three principal components showed clear separation of these groups in most cases. Modulators seem to affect TM7 bending at the conserved P7.50, which was expected. Surprisingly, PCA revealed interesting patterns of the helix bending at G7.42, next to the Y7.43 residue, which is an important part of the orthosteric binding site. The bending seems to be coupled with conformation of W7.40, especially in G_i1_-bound complexes, which is depicted in Fig. 8. Measurement of χ_1_ dihedral of this residue, depicted in Fig. 10, suggests that indeed it may be involved in the modulation mechanism, especially in case of the positive modulation by the R enantiomer of compound **1** and, to some extent, in NAM action of its S enantiomer. Notably, in all simulations of G_i1_-containing complexes, the dihedral value oscillates around − 180°/180° (which corresponds to the side chain protruding toward the membrane) except of the R1 compound simulation—in two of three replicas the dihedral is shifted to − 60° for the major fraction of time (which corresponds to the orientation towards TM1 and TM2). Interestingly, compound S1 was able to induce opposite shift in one of replicas (from − 60° to − 180° in replica 2). While similar effect was also observed in another simulation (R2_DG2), the latter was apparently of transient nature, while the former was permanent and persisted until the end of the simulation. In all other simulations W7.40 χ_1_ values seemed to be dependent on the type of G protein coupled, rather than on extracellular ligands. In G_i2_-bound complexes, the most pronounced effect in both PC1 and PC3 was the TM7 orientation in relation to neighbouring helices—both principal components indicate that PAM makes TM7 move closer to TM6, while NAM binding decreases distance between TM7 and TM2 (Fig. 9). Importantly, PCA indicates that in trajectories with PAM bound, Y7.53 usually points to the protein interior, while in NAM and SAM-bound complexes it tends to protrude toward the cytoplasm, regardless of the G protein type in a complex.

Conformation of TM6 was also suspected to play a role, based on the previously described RMSD values. However, only in complexes with G_i1_ protein bound different types of modulators were separated in the first two PCs to some extent. Effects of modulators on TM6 in G_i2_-bound systems in PCA were indistinguishable (data not shown).

Similarly to TM7, PCA of TM2 also provided interpretable results only in complexes with G_i1_. In contrast to TM6, in most simulations TM2 assumed stable, nearly identical conformations, and only the second and third replica yielded decreased PC1 values, corresponding to a different helix bending. This analysis has also shown that differences in interactions of modulators with W2.60 may be responsible for differences in signalling outcomes (data not shown).

### In vitro studies

#### Effects of compound 2 on the binding of orthosteric radioligand to D_2_ receptors

**Fig. 11:**
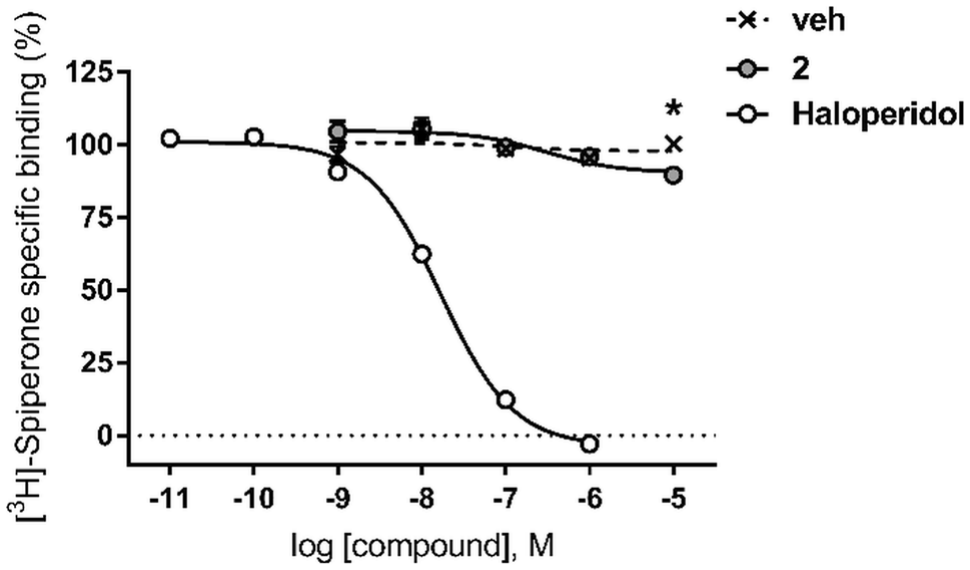
Competition radioligand binding assays of compound **2** at human D_2_ receptors. veh, vehicle (0.1% DMSO). The graph shows the data (mean ± SEM) of two independent experiments performed in duplicate. *P < 0.05 for vehicle vs. compound **2**, two-way ANOVA and Sidak's multiple comparisons test

To investigate the possible affinity of compound **2** for the orthosteric binding site of dopamine D_2_ receptor, the effects of compound **2** on the binding of the D_2_ orthosteric radioligand ([^3^H]-spiperone were investigated in vitro in competition radioligand binding assays on membranes from CHO-K1 cells stably expressing the human receptor. Displacement of the specific binding of [^3^H]-spiperone by compound **2** was only detectable at 10 µM concentration of the compound, the highest concentration assayed for solubility reasons. The % of displacement of the radioligand specific binding was (mean ± SEM, *n* = 2) 10.5 ± 0.8%, a small yet statistically significant effect (adjusted *P* value = 0.0373 for vehicle *vs.* compound **2**, two-way ANOVA (*F*_4,10_ = 4.269, *p* = 0.0285) and Sidak's multiple comparisons test) (Fig. 11). These results would be consistent with a barely detectable affinity of compound **2** for the orthosteric binding site of the receptor, whereas the reference D_2_ competitive antagonist haloperidol fully displaced the radioligand binding in the same conditions (Fig. 11). We obtained a *K*_i_ value for haloperidol of 8.85 nM, in good agreement with affinity values previously reported for this compound [42].

#### Activity of compound 2 in functional assays of cAMP signalling

**Fig. 12:**
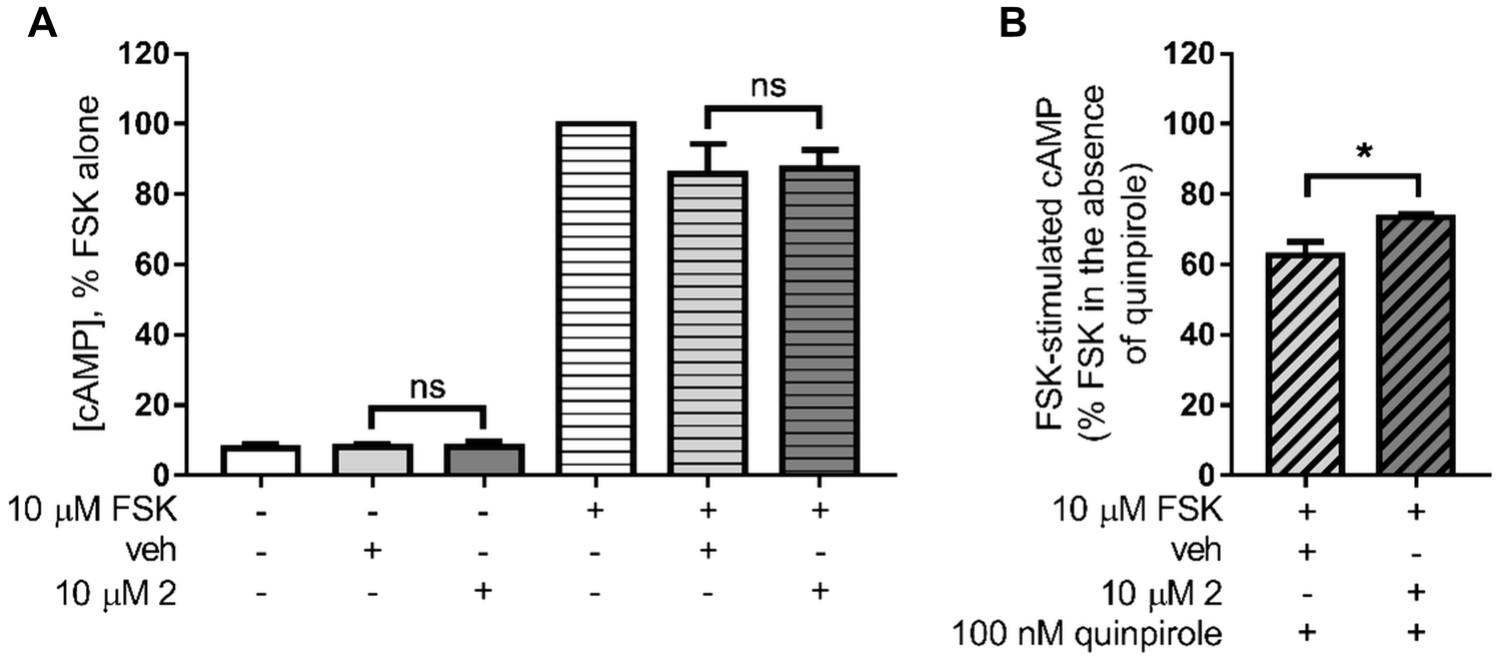
Functional assays of cAMP signalling for compound** 2** at human D_2_ receptors. **A** Cells stably expressing D_2_ receptors were exposed to vehicle (veh, 1% DMSO) or 10 µM compound **2**, and basal (no forskolin added) and 10 µM forskolin (FSK)-stimulated cAMP levels were determined (agonist mode). Data are expressed as % of FSK-stimulated cAMP in cells exposed to FSK alone (absence of vehicle or compound **2**). The graph shows the average (mean ± SEM) of normalized data from three (vehicle) to four (compound **2**) independent experiments performed in sextuplicate or greater. ns, no statistically significant difference for vehicle vs*.* compound **2** (adjusted *P* values = 0.9999 and 0.9720 in basal and forskolin-stimulated conditions, respectively; one-way ANOVA (*F*_3,10_ = 90.45, *p* < 0.0001) and Sidak's multiple comparisons test). Average cAMP concentrations in our assays were (mean ± SEM) 0.78 ± 0.31 nM and 8.91 ± 2.18 nM for basal and forskolin-stimulated cells, respectively (absence of vehicle or compound **2**) (not shown), 0.48 ± 0.04 nM and 4.97 ± 0.42 nM for basal and forskolin-stimulated cells, respectively (vehicle-treated cells), and 0.58 ± 0.03 nM and 7.46 ± 2.38 nM for basal and forskolin-stimulated cells, respectively (compound **2**-treated cells). **B** Effect of 100 nM quinpirole on forskolin (FSK)-stimulated cAMP production in the presence of vehicle (veh, 1% DMSO) or 10 µM compound **2**, in cells stably expressing D_2_ receptors. Data are expressed as % of FSK-stimulated cAMP in the absence of quinpirole at each condition (vehicle or compound **2**). The graph shows average (mean ± SEM) of normalized data from two (vehicle) to three (compound **2**) independent experiments performed in sextuplicate or greater. *p < 0.05, unpaired *t* test (*t*_*3*_ = 3.383, *p* = 0.0430)

Compound **2** was evaluated in functional assays of cAMP signalling at CHO-K1 cells stably expressing D_2_ receptors. Initial experiments aimed at detecting a possible direct agonistic or antagonistic activity of compound **2**. For this purpose, cells were incubated for 10 min with vehicle (1% DMSO) or 10 µM of compound **2** and, after that, 10 µM forskolin was added to the cells to stimulate cAMP signalling for 5 min (“agonist mode”) (Fig. 12A). Forskolin-stimulated cAMP levels were of the same magnitude in cells exposed to vehicle than in cells exposed to compound **2** (Fig. 12A), indicating no direct D_2_ agonist effect of the compound. We did not observe specific effects of compound **2** on basal (not forskolin-stimulated) cellular cAMP levels either (Fig. 12A). The possible D_2_ antagonistic effect of compound **2** was investigated in the presence of quinpirole, a selective D_2_ agonist. Cells were incubated for 5 min with vehicle (1% DMSO) or 10 µM of compound **2**, and after that 100 nM quinpirole was added for 10 min before 5 min stimulation with 10 µM forskolin (“antagonist mode”) (Fig. 12B). Under these conditions, 100 nM quinpirole reduced forskolin-stimulated cAMP levels to 62.5 ± 4.0% in the presence of vehicle and to 73.5 ± 0.9% in the presence of 10 µM compound **2** (*t*_*3*_ = 3.383, *p* = 0.0430, unpaired *t* test) (Fig. 12B). This modest yet statistically significant antagonism of quinpirole response by compound **2** could be related to the low D_2_ affinity of the compound detected in our radioligand binding assays. Higher concentrations of the compound were not tested in cAMP assays due to solubility limitations and because cell toxicity could not be ruled out based on the cytotoxicity data available (see Fig. S12 in the Supplementary Information).

Upon these in vitro findings and in the light of our in silico results, we aimed at investigate the possible functional consequences of the interaction of compound **2** with allosteric binding sites of the receptor. Hence, we carried out a detailed characterization of the effects of compound **2** on the response (inhibition of forskolin-stimulated cAMP production) of the orthosteric endogenous agonist dopamine in cAMP assays. In these experiments, we employed longer assay incubation times, which might favour the possible interaction of the compound with allosteric binding sites at the receptor.

**Fig. 13:**
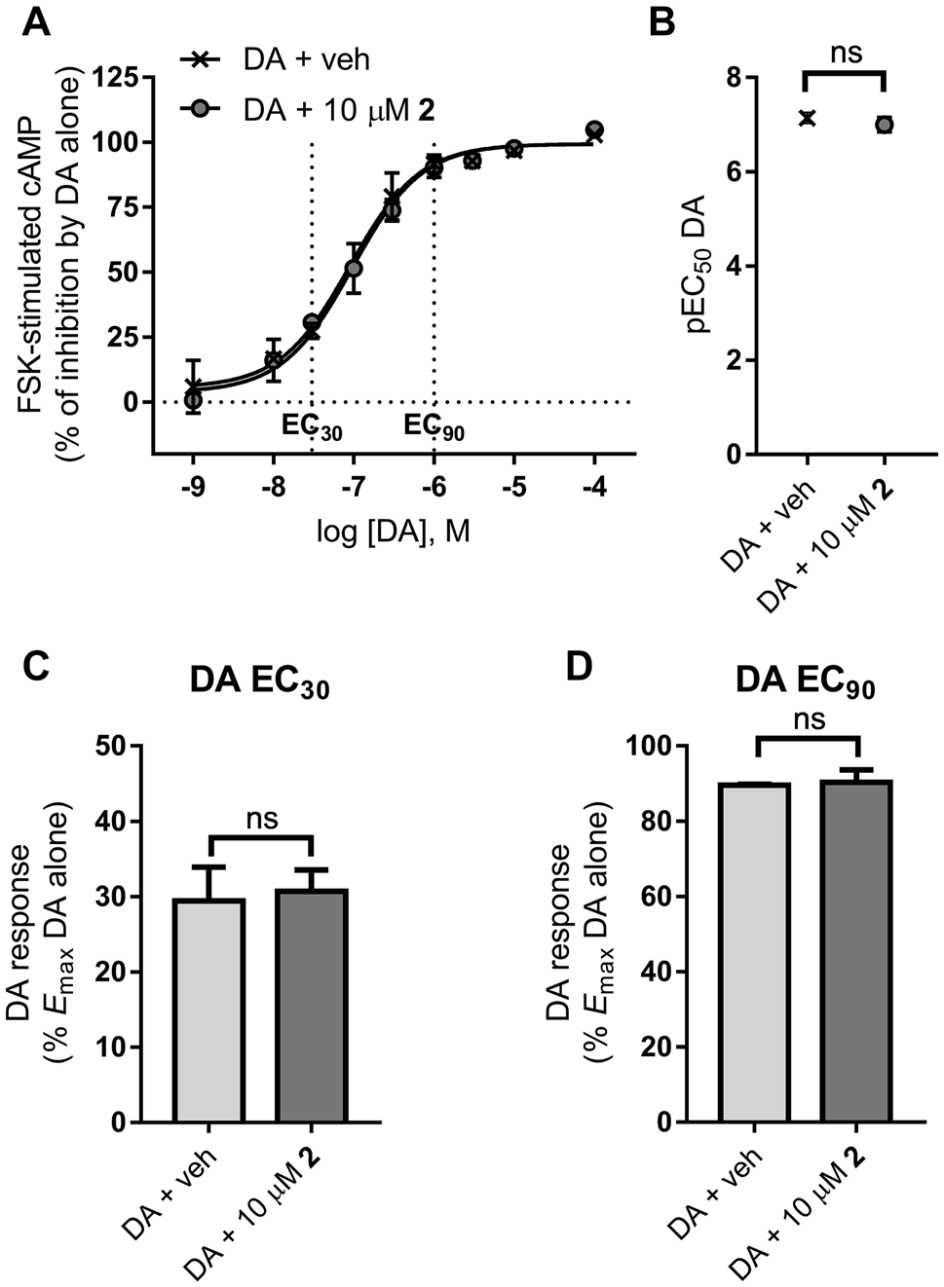
Effects of compound **2** on dopamine response (inhibition of forskolin-stimulated cAMP production) in functional assays of cAMP signalling at human D_2_ receptors. Cells stably expressing D_2_ receptors were incubated for 1 h in the presence of vehicle and/or ligands and 10 µM forskolin. **A** Dopamine (DA) concentration–response curves in the presence of vehicle (veh, 1% DMSO) or 10 µM compound **2**. Response is expressed as % of the maximal inhibition elicited by dopamine in the absence of vehicle or compound **2** (“DA alone”). The graph shows average (mean ± SEM) of normalized data from two (vehicle) to four (compound **2**) independent experiments performed in sextuplicate. **B** Potency (pEC_50_) of dopamine in the presence of vehicle or 10 µM compound **2** in these cAMP assays. The graph shows average (mean ± SEM) pEC_50_ values from the individual experiments considered in **A**). ns, no statistically significant difference for vehicle vs*.* compound **2** (*p* > 0.05, unpaired *t* test) (*t*_*4*_ = 0.669, *p* = 0.5401). **C**, **D** Bar graphs showing dopamine response in the presence of vehicle or compound **2**, at dopamine concentration data points close to dopamine EC_30_ (**C**) or EC_90_ (**D**) as extracted from the dopamine concentration–response curves from the individual experiments considered in A). ns, no statistically significant difference for vehicle vs*.* compound **2** (*p* > 0.05, unpaired *t* test) (*t*_*4*_ = 0.2609, *p* = 0.8071; *t*_*4*_ = 0.1521, *p* = 0.8865, for dopamine EC_30_ (**C**) and dopamine EC_90_ (**D**), respectively). Average cAMP concentrations in the absence of vehicle or compound **2** (“DA alone”) were (mean ± SEM) 0.36 ± 0.04 nM, 21.5 ± 5.5 nM, and 5.01 ± 1.62 nM for basal (not forskolin-stimulated), forskolin-stimulated, and forskolin + maximal dopamine-stimulated cells, respectively (not shown)

Concentration–response curves of dopamine (1 nM–100 µM) were carried out in the presence of vehicle or 10 µM compound **2** (Fig. 13). Cells were incubated with vehicle and/or ligands and 10 µM forskolin for 1 h, following a protocol that has previously allowed the identification of positive allosteric modulators of D_2_ receptor [21]. As it occurred in cAMP assays using shorter incubation times, we did not observe direct effects of compound **2** either in basal (not forskolin-stimulated) or forskolin-stimulated cellular cAMP levels in the absence of dopamine. The average cAMP concentrations were 0.38 ± 0.07 nM and 15.6 ± 5.7 nM for basal and forskolin-stimulated cells, respectively in vehicle-treated cells, and 0.35 ± 0.06 nM and 18.7 ± 3.8 nM for basal and forskolin-stimulated cells, respectively, in compound **2**-treated cells (adjusted *P* values > 0.9999 and = 0.7798 for vehicle *vs.* compound **2** in basal and forskolin-stimulated conditions, respectively; one-way ANOVA (*F*_3,8_ = 10.27, *p* = 0.0041) and Sidak's multiple comparisons test) (not shown). Dopamine inhibited forskolin-stimulated cAMP production in a concentration-dependent manner similarly to that in the presence of vehicle or compound **2** (Fig. 13A), while compound **2** did not affect the potency of dopamine in these assays (pEC_50_ (mean ± SEM) = 7.14 ± 0.11 and 7.00 ± 0.13 for vehicle and compound **2**, respectively) (*t*_*4*_ = 0.669, *p* = 0.5401, unpaired *t* test) (Fig. 13B).

At dopamine concentration in its EC_20_ to EC_40_ range, which would afford maximal sensitivity for detection of enhancing effects by positive allosteric modulators [54], no PAM activity of compound **2** was observed (Fig. 13A, C). Dopamine response (% *E*_max_ of dopamine in the absence of vehicle or compound **2**) was (mean ± SEM) 29.4 ± 4.5 and 30.7 ± 2.8 for vehicle and compound **2**, respectively (*t*_*4*_ = 0.2609, *p* = 0.8071, unpaired t test) (Fig. 13C). Similarly, at dopamine submaximal concentrations (EC_80_–EC_90_), optimal for measuring dampening effects by negative allosteric modulators [54, 55], no NAM effect on dopamine response by compound **2** was detected. Dopamine response (% *E*_max_ of dopamine in the absence of vehicle or compound **2**) was (mean ± SEM) 89.5 ± 0.3 and 90.3 ± 3.4 for vehicle and compound **2**, respectively (*t*_*4*_ = 0.1521, *p* = 0.8865, unpaired *t* test) (Fig. 13D). A possible mode of action of allosteric modulators is to alter the dissociation kinetics of an orthosteric ligand. Yet with the limitation of possible probe dependence in allosteric effects. it should be mentioned at this point that radioligand binding kinetic experiments at D_2_ receptors revealed no effect of compound **2** on the dissociation rate constant (*k*_off_) of [^3^H]-spiperone (see Fig. S13 in the Supplementary Information). Therefore, following an experimental design that would favour the interaction of allosteric modulators with the allosteric binding site predicted by our in silico studies, compound **2** did not affect the response to the orthosteric agonist as it is expected for a silent (or neutral) allosteric modulator (SAM) [55], whereas a PAM effect was observed for compound **1R** and a weak NAM effect for **1S** by an independent group following the same experimental protocol to the best of our knowledge [21].

## Discussion

D_2_ receptor has been an important model system for understanding allosteric modulation of GPCR signalling [14, 56, 57] and an important target for typical [58] and atypical [59, 60] antipsychotics used to treat schizophrenia [61] and for therapeutic strategies in Parkinson’s disease [62–64], as well as a target of interest in Alzheimer’s disease [65, 66]. The aim of the present work was to investigate the effect of four ligands as allosteric modulators of the constructed full models of the dopamine D_2L_ receptor in complex with a natural agonist, dopamine, and with G protein (with Gα_i1_ or Gα_i2_ subunit). Although models of the dopamine D_2_ receptor in active conformation with or without the respective G protein are already available in the literature [67–69], this is, to our best knowledge, the first time the allosteric modulators were docked to full D_2L_ isoform, including ICL3 loop.

Compound **1**, reported by Wood et al., was the basis to design and synthesize compound **2** [21] to check its possible allosteric effect on dopamine D_2_ receptor. Detailed in vitro studies of this compound in a form of a racemate, which included radioligand binding assay, functional studies, and kinetic assay, indicated that compound **2** may be a SAM of the receptor. It should be stressed that the probe dependence effect cannot be excluded in this case [70].

The models of dopamine D_2_ receptor used in our research turned out to be very similar to the corresponding X-ray structure, published relatively recently, PDB ID: 6VMS [25]. The observed RMSD may be due in part to the fact that the crystal structure contains a synthetic agonist bromocriptine. In addition, the crystal structure is a single frame of the studied crystal which does not reflect the actual dynamics of the protein, the quality of the model can therefore be regarded as satisfactory.

The availability of structural data about the possible allosteric sites of GPCRs is crucial for structure-based drug design [71, 72]. The comparison of available X-ray and electron microscopy structures makes it possible to distinguish the most common binding sites for GPCRs allosteric modulators. Firstly, in the transmembrane bundle, the allosteric sites can be classified into three groups: [73] (i) at the extracellular side of the receptor, (ii) at the central 7TM helical bundle and (iii) at the intracellular side of the receptors [72]. Recently, Xiao et al. [74] described a dopamine D_1_–G_s_ complex simultaneously bound to dopamine in the orthosteric site and the PAM LY3154207 in an intracellular allosteric pocket [75]. This allosteric binding pocket at D_1_ receptor was confirmed by Zhuang et al. [76]. Such intracellular allosteric binding sites are also known for other GPCRs (class A chemokine CCR2 [77], CCR7 [78] and CCR9, [79] and β_2_AR receptors [80]) and are possible but have been not experimentally verified yet for D_2_-like receptors. Moreover, the allosteric sites at the receptor–lipid bilayer interface are situated at various faces of the receptor [72].

There are few literature reports on molecular docking and molecular dynamics simulations of allosteric modulators of dopamine D_2_ receptor [45, 47–49]. In these studies, SB269652, a bitopic ligand which interacts with both the orthosteric binding site and a secondary binding pocket in both D2R and D3R, was described as a NAM. The secondary binding pocket was identified between TM2 and TM7, similarly to the allosteric site in our work. Importantly, selected residues of dopamine D_2_ receptor were mutated to indicate which of them affect binding and functional properties of SB269652. It was also found that SB269652 exerts allostery across the D2R dimer. A similar secondary binding pocket has been recently found for dopamine D_3_ receptor [43]. As there is no structural similarity between compounds **1** and **2** and SB269652 and similarly acting compounds [81], in particular compounds **1** and **2** cannot be considered bitopic ligands, their allosteric effect results rather from the interaction with the receptor monomer, not a dimer.

In this work, we used molecular docking approach to identify the region of the receptor that is most likely involved in the binding of allosteric modulators. The position of the allosteric pocket depended on the type of model used. In DG1, all modulators bound deeper into the receptor, just above dopamine, while ligands in the DG2 model bound closer to the extracellular part of the receptor. During molecular dynamics simulations, ligands bound to DG2 migrated into lower binding pockets.

The examined RMSD values for individual helices allowed determining the most dynamic receptor structures. In particular, TM5, TM6 and TM7 movements turned out to be significant in the MD study of allosteric modulation of the ligands used. The results obtained in this study explain the phenomena of positive modulation with the R1 ligand in DG1 and DG2. Because of rotameric transitions of Y5.58 and Y7.53, their side chains can be placed within the space emptied by the outward movement of TM6. Thus, rearrangement of these residues appears to stabilize the receptor in its active conformation by structural water-mediated hydrogen bond network [82, 83]. Whereas the hydrogen bond between Y5.58 and Y7.53 remained stable throughout the simulation with R1, modulator S1 caused a larger fluctuation and increase in the distance between these residues. Furthermore, in the case of S1, the organization of TM5, TM6 and TM7 differs significantly. We also examined the behaviour of several microswitches, which are important for the GPCR activation process. We analysed changes in the dihedral angle of the conserved W6.48 [83–86], F6.44 (called transmission switch) [87–89], Y7.53 of NPxxY motif [90], and H6.55 (a crucial residue for dopamine D_2_ receptor activation [91, 92]). The α5 helix of the Gα subunit is a critical region for the receptor-mediated and basal activity [93, 94]. Therefore, we used MD simulations to investigate how the studied modulators affect the interactions with the Gα_i1_ and Gα_i2_ proteins and receptor activation processes. This observation indicates that changes in the position of α5-Gα helix are slight, but sufficient to determine the type of modulation. The R1 modulator stabilizes the C-terminus of Gα_i_ proteins in a position into the binding pocket higher than the S1 modulator. In contrast to R1 and S1, in this study, we did not observe significant differences in the action of R2 and S2 compared to the dopamine receptor simulations.

Finally, we performed principal component analysis to detect—in a statistical manner—the relationships between modulator structures and receptor conformation. The most pronounced changes were found in TM7 with helix bending at P7.50 and G7.42 situated near Y7.43, an important component of the orthosteric binding site. Importantly, PCA indicates that conformation of the Y7.53, which is a part of the NPxxY motif and conserved in Class A GPCRs, is affected by the enantiomers of compound **1** in opposite ways, which is in line with their signalling outcomes observed in in vitro assays and therefore serves as a validation of the in silico part of the study. Notably, in all these analyses, conformations of compound **2** complexes assume intermediate conformations, in between extremes explored by NAM and PAM enantiomers of compound **1**, which, together with in vitro results, supports the conclusion that compound **2** does not affect conformational space explored by the receptor upon binding, i.e. behaves like SAM. Moreover, PCA allows drawing more general conclusions on mechanisms employed by all the investigated compounds. There is apparent difference in the receptor response depending on the G_i_ protein subtype bound. While differences revealed by the PCA of all trajectories in the common subspace (Fig. S11) can be considered as an artefact originating in modelling and equilibration steps, different sensitivity of TM2 and TM6 to the modulators in G_i1_ and G_i2_-bound complexes may indicate possible allosteric functional selectivity, which points to new directions for further studies of these modulators. Additionally, PCAs suggest a role of particular residues that are possibly involved in the allosteric signal transmission. In particular, W7.40 conformation seems to be affected by the presence of the PAM compound (Fig. 10).

In summary, the in silico results obtained in this work show that the R1 and S1 are able to bind in an allosteric site of the D2R and thereby promote conformational changes of helical bundle that can be associated with the transition towards an active or inactive state, respectively. In case of R2 and S2, the binding to the D2R in the allosteric pocket is devoid of significant influence on the receptor activation mechanism which is supported by in vitro data. Our work indicates that the allosterism at GPCRs, in particular at dopamine D_2_ receptor, is governed by subtle structural and stereochemical ligand modifications, which significantly hampers the efforts to obtain a series of modulators to study their SAR.

## Supplementary Information

Below is the link to the electronic supplementary material.

Supplementary file1 (DOCX 8788 KB)

## Abbreviations

DG1: D2LONG receptor in complex with G_i1_
DG2: D2LONG receptor in complex with G_i2_
ECL: Extracellular loop
GPCRs: G protein-coupled receptors
ICL: Intracellular loop
MD: Molecular dynamics
NAM: Negative allosteric modulator
PAM: Positive allosteric modulator
PCA: Principal component analysis
R1: (1,3-Benzothiazol-2-yl(2-methyl-2,3-dihydro-indol-1-yl)methanone, enantiomer R
R2: (4-Methoxy-1-methyl-*1H*-indol-2-yl)(2-methyl-2,3-dihydro-*1H*-indol-1-yl)methanone, enantiomer R
RMSD: Root-mean-square deviation
S1: (1,3-Benzothiazol-2-yl(2-methyl-2,3-dihydro-indol-1-yl)methanone, enantiomer S
S2: (4-Methoxy-1-methyl-*1H*-indol-2-yl)(2-methyl-2,3-dihydro-*1H*-indol-1-yl)methanone, enantiomer S
SAM: Silent allosteric modulator
TM: Transmembrane
