# Supplementary material for: Allosteric modulation of dopamine D2L receptor in complex with Gi1 and Gi2 proteins: the effect of subtle structural and stereochemical ligand modifications
Source: Pharmacol Rep. 2022 Jan 22;74(2):406–24. doi: 10.1007/s43440-021-00352-x (PMC8964653; doi:10.1007/s43440-021-00352-x)

Supplementary Information

## Allosteric modulation of dopamine D_2L_ receptor in complex with G_i1_ and G_i2_ proteins - the effect of subtle structural and stereochemical ligand modifications

Justyna Żuk^1^, Damian Bartuzi^1^, Andrea G. Silva^2^, Monika Pitucha^3^, Oliwia Koszła^1^, Tomasz M. Wróbel^1^, Dariusz Matosiuk^1^, Marián Castro^2^ and Agnieszka A. Kaczor^1,4^*

*^1^ Department of Synthesis and Chemical Technology of Pharmaceutical Substances with Computer Modeling Laboratory, Faculty of Pharmacy, Medical University of Lublin, 4A Chodźki St., PL-20093 Lublin, Poland*

*^2^ Department of Pharmacology, Universidade de Santiago de Compostela, Center for Research in Molecular Medicine and Chronic Diseases (CIMUS), Avda de Barcelona, Santiago de Compostela, E-15782, Spain*

*^3^ Independent Radiopharmacy Unit, Faculty of Pharmacy, Medical University of Lublin, 4A Chodźki St., PL-20093 Lublin, Poland*

*^4^ School of Pharmacy, University of Eastern Finland, Yliopistonranta 1, P.O. Box 1627, FI-70211 Kuopio, Finland*

******* *Correspondence: agnieszka.kaczor@umlub.pl*

**Keywords:** dopamine D_2_ receptor; GPCRs; molecular dynamics; molecular switches; negative allosteric modulators, positive allosteric modulators

**Supplementary *in silico* description**

**Fig. S1-S11**

**Supplementary *in vitro* Methods**

**Supplementary *in vitro* Results**

**Fig. S12-S15**

**Similarity of compound 1 and 2 to the known dopamine D_2_ receptor ligands available in the CHEMBL database.**

**Table S1.**

**Possible pharmacological properties of compounds 1 and 2 predicted by PASS software.**

**Table S2.**

**Spectra for compound 2**

**Supplementary *in silico* description**

R1 bound to DG1

Fig. S1 A presents the time evolution of χ1 dihedrals of W6.48 of the CWxP motif, F6.44, H6.55 and Y7.53 of the NPxxY motif.

The most dynamic changes have been observed for W6.48, Y.7.53 and H6.55. The most stable values were seen at F6.44, with dihedral oscillating around -90 degrees. The Y.7.53 and W6.48 side chains adopt two distinct dihedral values, referred to as states 1, 2 (B and C on Fig. S1, respectively). In state 1, the W6.48 residue (oscillating around -120 degrees) points into the direction of TM7, while state 2 shows the side chain oriented towards the receptor interior. In all replicas W6.48 tends to oscillate around -60 degrees, so the state 2 represents favourable conformation of tryptophan. The side chain of Y7.53 in state 1 (oscillating around -60 degrees) points toward the interior of the receptor. In the state 2 (oscillating around +100 degrees) the tyrosine moves more outward pointing toward TM2. Furthermore, the analysis of the surrounding residues showed that in this position a water-mediated interaction forms between Y5.58-Y7.53. This interaction between tyrosines contributes to the stabilization of the receptor in the active state. At the end of the simulation the C_α_ atoms of Y5.58 and Y7.53 are separated by <17,5 Å (B on Fig. S1), while in simulation of dopamine_DG1 complex tyrosines are separated by >18 Å (not shown).

R1 bound to DG2

Fig. S2 A shows the time evolution of dihedral angles of W6.48, F6.44, H6.55 and Y7.53 that undergo side chain conformational transitions in R1_DG2. W6.48 adopts values of −60 degrees for most of the simulation time. This conformation is stabilized by hydrogen bond with S5.43 and a π -π stacking interaction with Y5.48, which constitutes a stable bridge between TM5 and TM6 (Fig. S2-B). The studied dihedral angle of H6.55 shows significant flexibility and adopts three main distinct states, namely state 1, 2 and 3, oscillating around +180/+120/-60 degrees, respectively (Fig. S2-C). Residue F6.44 showed frequent transitions between 1 and 2 states (+150/-150 degrees, respectively). The side chain of F6.44 in state 1 points toward TM5, which results in forming interaction with T5.54, while in the less stable state 2 the side chain of phenylalanine interacts with N7.45 of TM7 (Fig. S2D). Conformation of Y7.53 residue showed rather low variations with χ1 dihedral values, oscillating around +120 degrees, forming water-mediated hydrogen bond with Y5.58 (Fig. S2E). The distances between Y5.58 and Y7.53 significantly decreased at the end of MD simulations.

S1 bound to DG1 and DG2

The time evolution of χ1 dihedrals of W6.48, F6.44, H6.55 and Y7.53 for S1_DG1 and S1_DG2 complexes is shown on Fig. S3 A and S4 A, respectively. The χ1 of W6.48 adopts values of −60° most of the time. The χ1 dihedral of H6.55 showed significant flexibility. In the second replica of S1_DG2 it found an energetically favourable conformation after 276 ns, remaining around 180° to the end of the simulation. Residue F6.44 showed variations between state 1 and 2 (+100°/-100°, respectively). In the state 1, in simulations with S1_DG2 complex, the side chain of phenylalanine forms π -π interaction with Y7.53, which stabilizes the tyrosine conformer of a dihedral angle of -150°, and therefore disrupts the network of water-mediated hydrogen bonds with Y5.58 (Fig. S6, **C**). We also observe increased distance between Cα atoms of Y5.58 and Y7.53 (Fig. S3 A and S4 A).

R2 and S2 bound to DG1 and DG2

Fig. S5 and S6 show side chain torsion angles for R2_DG1 and S2_DG1, during 1 μs MD simulations. Apart from initial motility, the W6.48 switch oscillated around -60°. The χ1 dihedral of H6.55 showed flexibility oscillating around +180°. The high shift and oscillation of the residues F6.44 and Y7.53 is observed. The side chain of F6.44 showed transitions between +100° and -100°, with the latter being more stable (Fig. S5 and S6 C). The side chain of Y7.53 toggle switch undergoes oscillation around -100°/+60°. The continuous water channel is formed inside the receptor, but, as in the dopamine_DG1 complex (not shown), the network of water-mediated interactions between Y.5.58 and Y7.53 did not form (Fig. S5 and S6 D).

The action of molecular switches for R2 and S2 modulators bound to D2 in complex with G_i2_ are shown on Fig. S7 and S8. The studied χ1 dihedral of W6.48 is stabilized at about -60 degrees in all simulations. The χ1 dihedral of H6.55 showed significant flexibility oscillating between +180° and +60°. In the conformation adopting the dihedral value of +60°, histidine forms a hydrogen bond with S5.43 in R2_DG2 complex and π -π interaction with F6.51 in S2_DG2 (Fig. S7 and S8 B). Residue F6.44 showed high variations oscillating between values +100°/-100°. Y7.53 was stable during the simulations. This conformation allows water-mediated interactions with Y5.58 (Fig. S7 and S8C) as in the dopamine_DG2 complex (not shown).

**Contact maps analysis**

The R1 modulator bound to DG1 is positioned directly above the dopamine and this position was stable during 1 µs of MD simulations. The R1 bound to DG2 moved into deeper pocket and closer to dopamine. Specifically, the 2-methyl-2,3-dihydroindol-1-yl group is situated between Y7.35, W100 (ECL1) and I184 (ECL2). Moreover, in three replicas of R1_DG1 and two replicas of R1_DG2 residues F3.28 and D3.32 form a trough to the benzothiazole group of the modulator (Supplementary Information, Figure S9, B and C). The role of F3.28 and D3.32 is worth noting as it appears on all the maps with R1. In the case of S1 modulator, proximity to S5.42, S5.43 and S5.46 seems to play a role. The S1 modulator may weaken interactions of these residues with dopamine. Moreover, in one replica of S1_DG2 the hydrogen bonds between dopamine and the serines broke with simultaneous rotation of orthosteric ligand and formation of a hydrogen bond between its metahydroxyl group and D3.32 (Supplementary Information, Figure S9, D). Results for R2 show favourable position of [4-methoxy-1-methylindole](https://www.sigmaaldrich.com/catalog/product/aldrich/259055?lang=en&region=US) substituent very close to W100 (ECL1) and Y7.35. In two replicas of R2_DG1 and R2_DG2 complexes this group is situated directly between those residues, forming a three-layered aromatic stack (Supplementary Information, Figure S10, B and C). The results of MD simulations showed that S2 took favourable positions near H6.55, Y7.35 and W100 (ECL1) and interacted with these residues by π -π interaction and/or hydrogen bond with carbonyl oxygen of the ligand (Supplementary Information, Figure S10, D and E). Furthermore, residue A185 of ECL appears in all R2 and S2 contact maps.


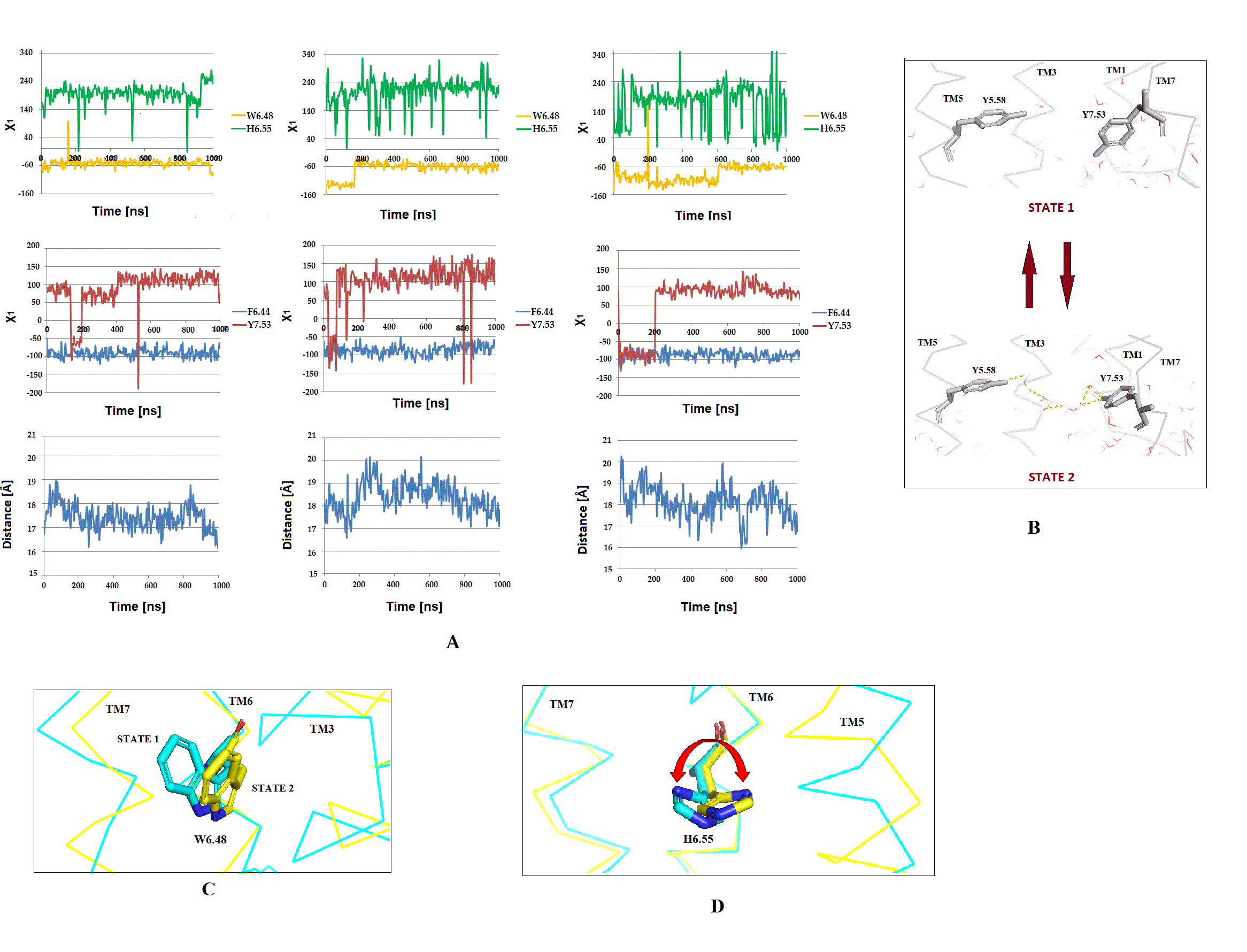


**Figure S1. A**: side chain torsion angle changes of W6.48, H6.55, F6.44 and Y7.53 and distance of Y5.58 and Y7.53 for R1_ DG1 complexes in three replicas during 1μs MD simulations. **B**: the side chains of Y7.53 in relation to Y5.58 visualized as grey sticks. A (water-mediated) hydrogen bond between residues Tyr5.58 and Tyr7.53 (yellow dashes) was only observed in state 2. **C**: two conformational state of W6.48 are depicted as cyan and yellow sticks with blue nitrogen and red oxygen atoms. **D**: residue H6.55 in two conformational states visualized as cyan and yellow sticks with blue nitrogen atoms. The direction of changes is provided as a red arrow. The helices in ribbon representation. Hydrogen atoms of residues omitted for clarity.


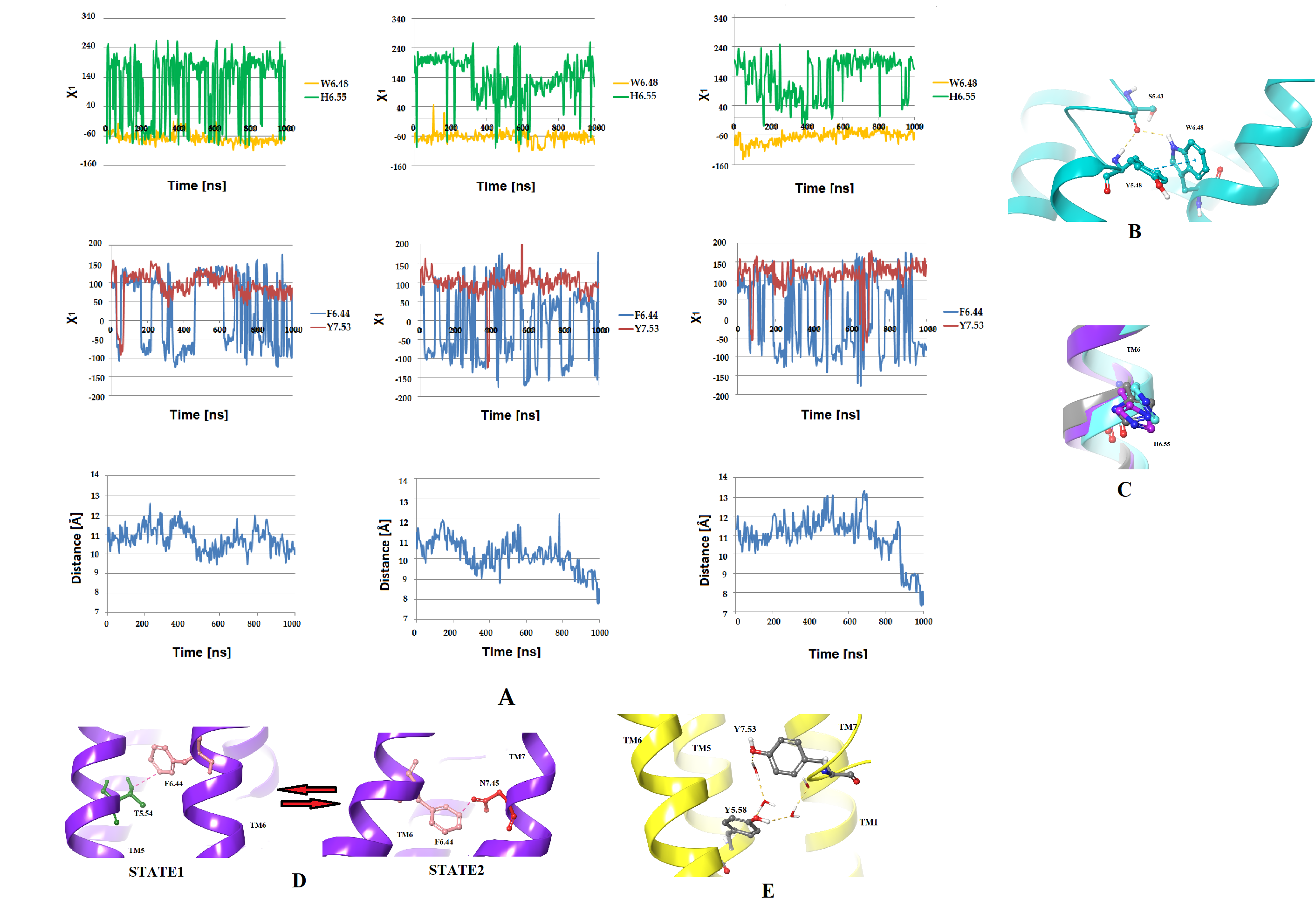


**Figure S2. A**: side chain torsion angle changes of W6.48, H6.55, F6.44 and Y7.53 and distance of Y5.58 and Y7.53 for R1_ DG2 complexes in three replicas during 1μs MD simulations. **B:** mutual positions of S5.43, Y5.48 and W6.48, residues shown as cyan sticks. **C**: residue H6.55 in two conformational states visualized as cyan and purple sticks. **D:** mutual positions of F6.44 (pink sticks) and T5.54 (green sticks) or N7.45 (red sticks) in two conformational states. **E**: the side chains of Y7.53 in relation to Y5.58 visualized as grey sticks. A (water-mediated) hydrogen bond between residues Tyr5.58 and Tyr7.53 (yellow dashes) was only observed in state 2.


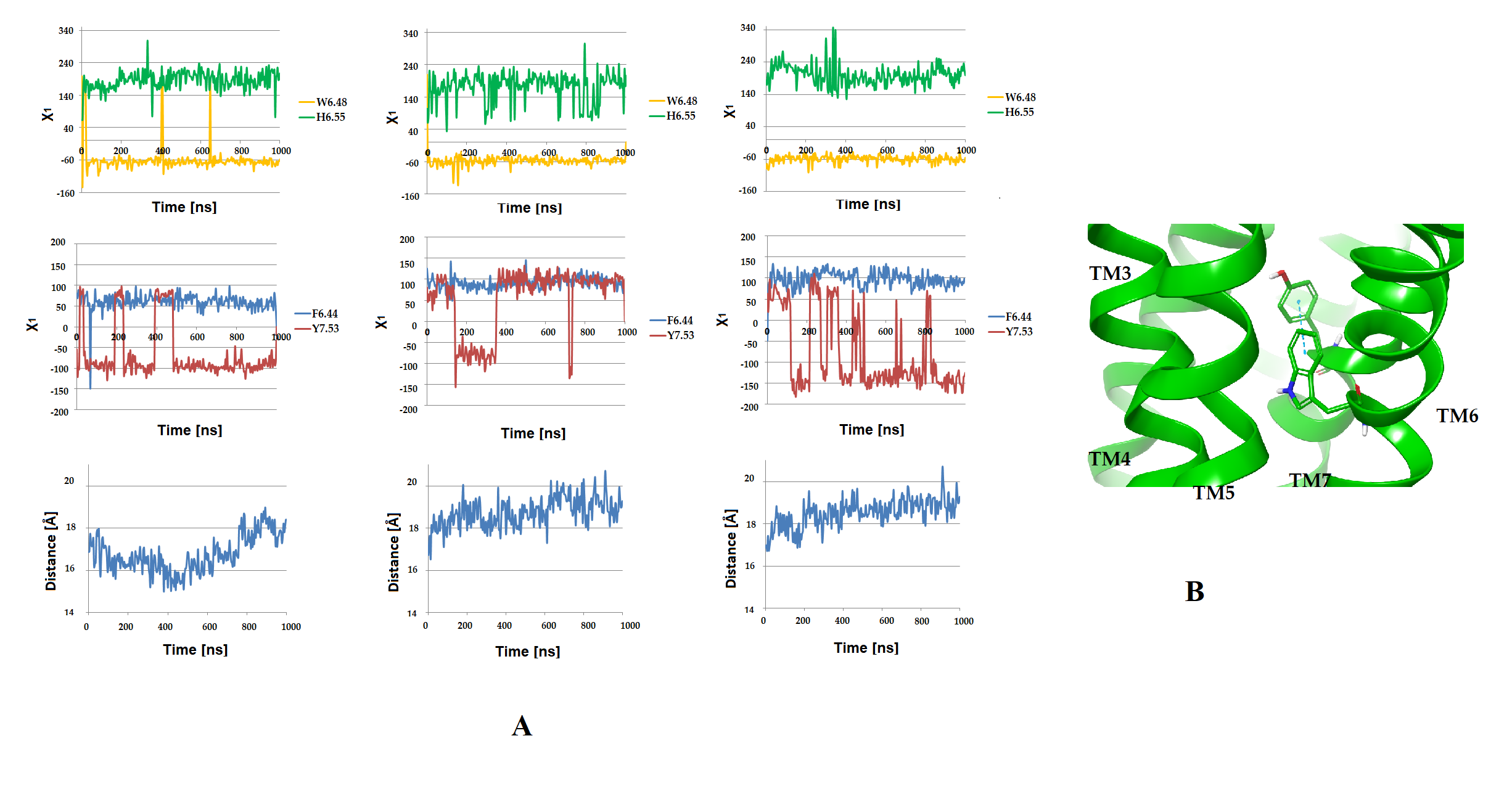


**Figure S3. A**: the side chain torsion angle changes of W6.48, H6.55, F6.44 and Y7.53 and distance of Y5.58 and Y7.53 for S1_ DG1 complexes in three replicas during 1μs MD simulations. **B**: the unstable conformational state of W6.48 interacting with F6.52 (green dashes). Residues are depicted as green sticks with blue nitrogen and red oxygen atoms. **C**: residue F6.44 forming π -π interaction (blue dashes) with Y7.53 and N7.45. Residues in a green sticks representation.


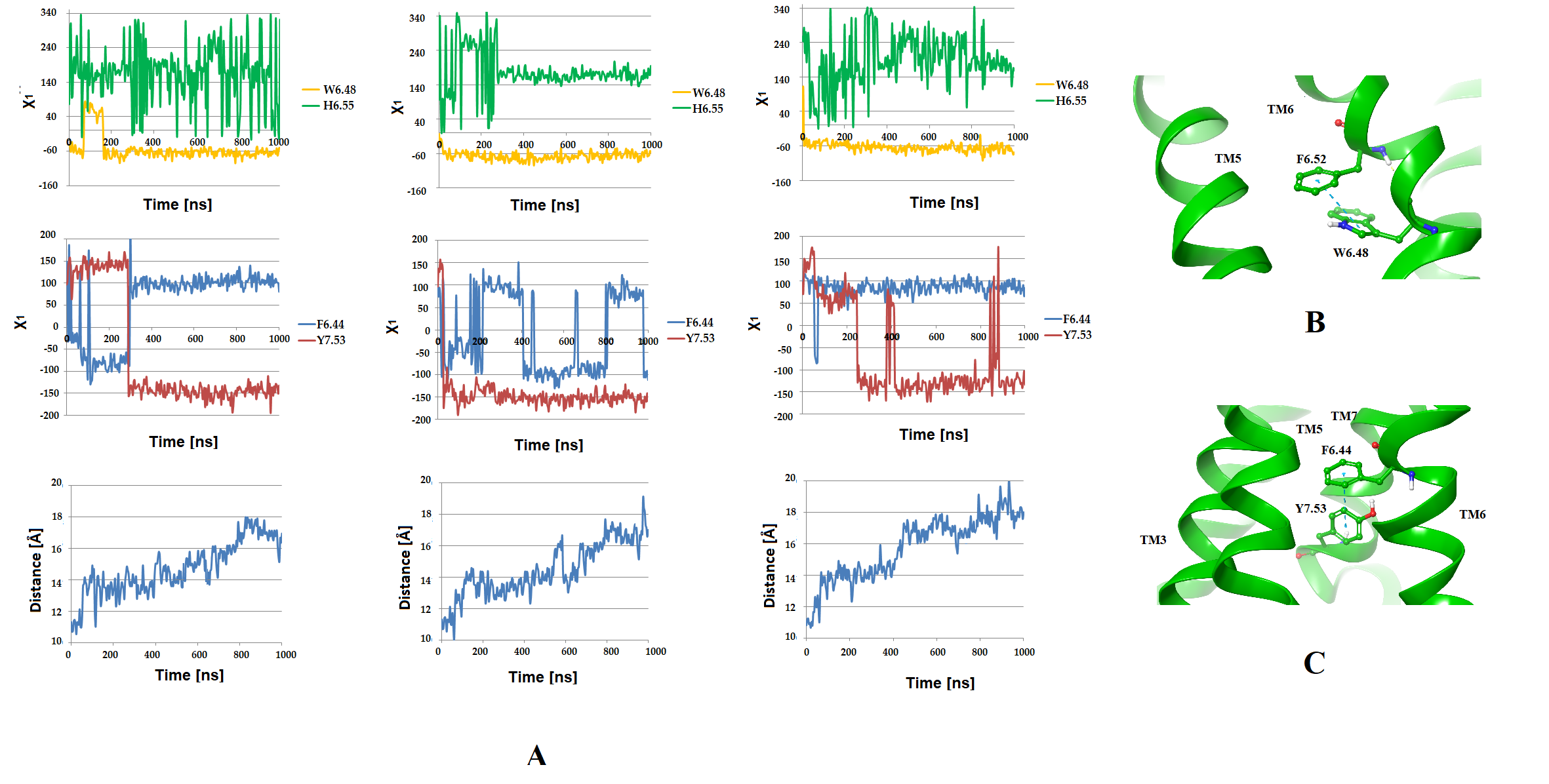


**Figure S4. A**: the side chain torsion angle changes of W6.48, H6.55, F6.44 and Y7.53 and distance of Y5.58 and Y7.53 for S1_ DG2 complexes in three replicas during 1μs MD simulations. **B**: the unstable conformational state of W6.48 interacting with F6.52 (green dashes). Residues are depicted as green sticks with blue nitrogen and red oxygen atoms. **C**: residue F6.44 in a green sticks representation forming π -π interaction (blue dashes) with Y7.53.


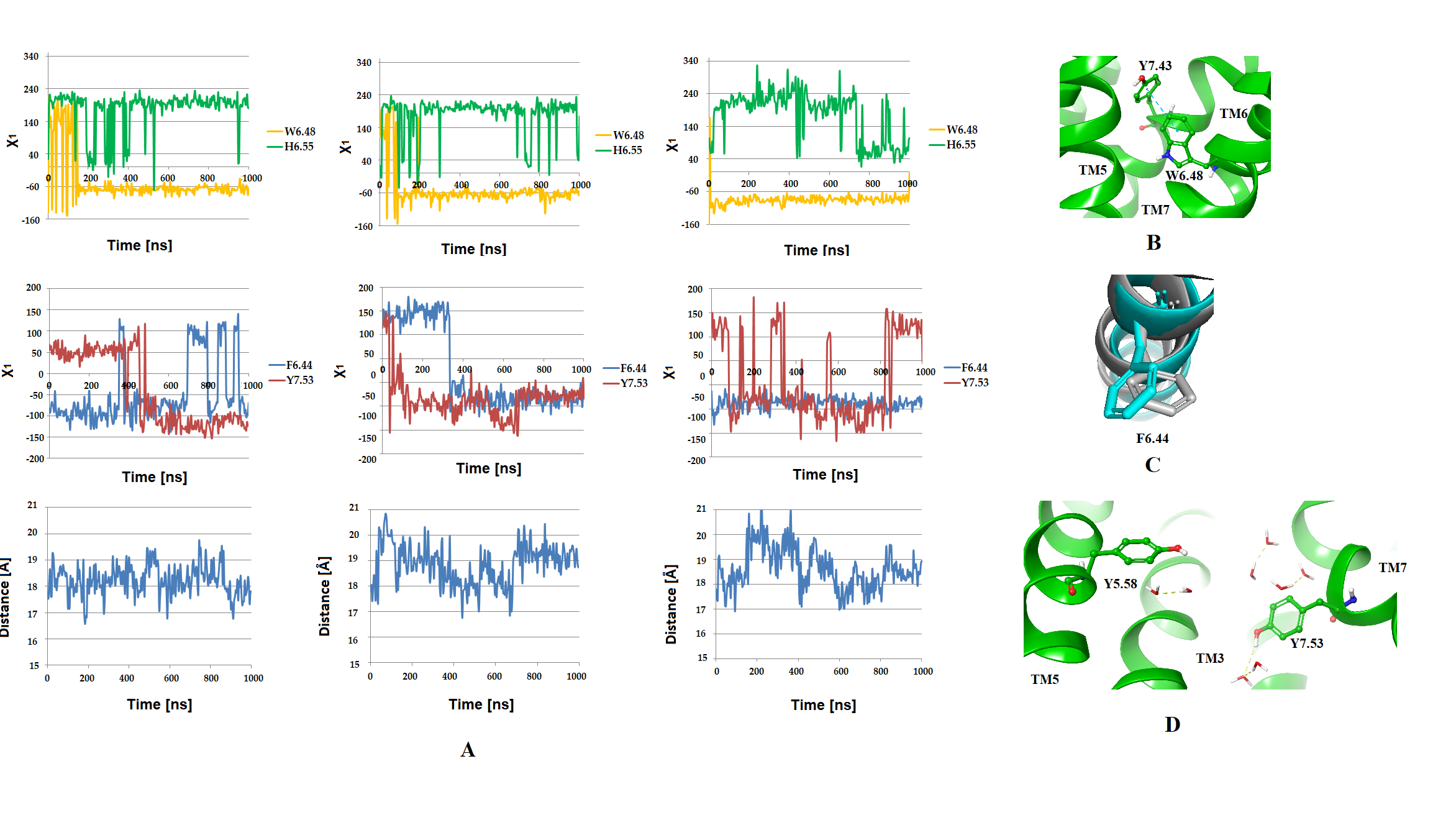


**Figure S5. A**: the side chain torsion angle changes of W6.48, H6.55, F6.44 and Y7.53 and distance of Y5.58 and Y7.53 for R2_DG1 complexes in three replicas during 1μs MD simulations. **B**: the unstable conformational state of W6.48 interacting with Y7.43 (green dashes). Residues are depicted as green sticks with blue nitrogen and red oxygen atoms. **C**: residue F6.44 in a sticks representation. The conformation in grey color is more stable. **D**: the side chains of Y7.53 in in relation to Y5.58 visualized as grey sticks with blue nitrogen and red oxygen atoms. A (water-mediated) hydrogen bonds showed as yellow dashes.


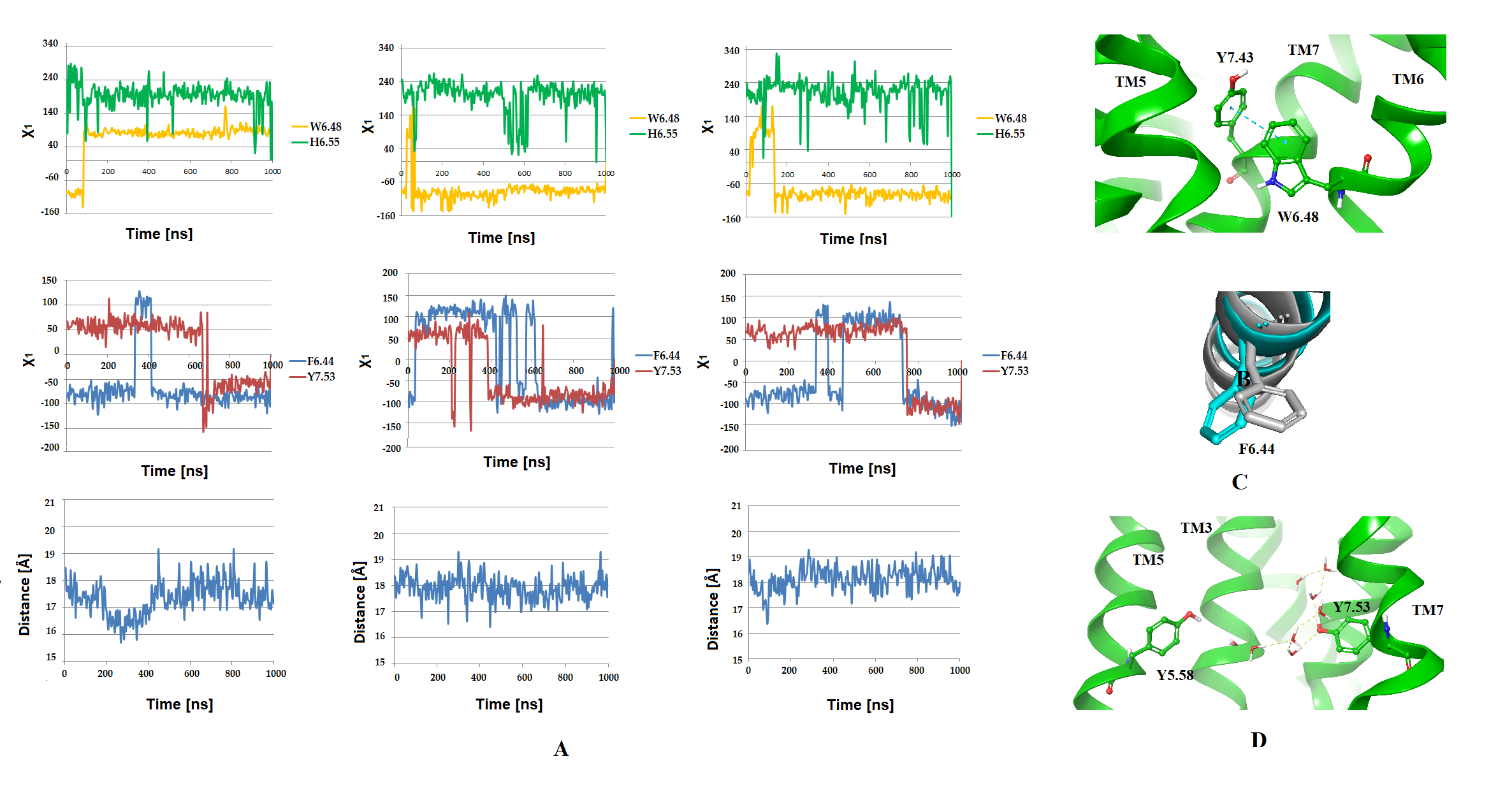


**Figure S6. A**: the side chain torsion angle changes of W6.48, H6.55, F6.44 and Y7.53 and distance of Y5.58 and Y7.53 for S2_DG1 complexes in three replicas during 1μs MD simulations. **B**: the unstable conformational state of W6.48 interacting with Y7.43 (green dashes). Residues are depicted as a green sticks with blue nitrogen and red oxygen atoms. **C**: residue F6.44 in a sticks representation. The conformation in grey color is more stable. **D**: the side chains of Y7.53 in in relation to Y5.58 visualized as grey sticks with blue nitrogen and red oxygen atoms. A (water-mediated) hydrogen bonds showed as yellow dashes.


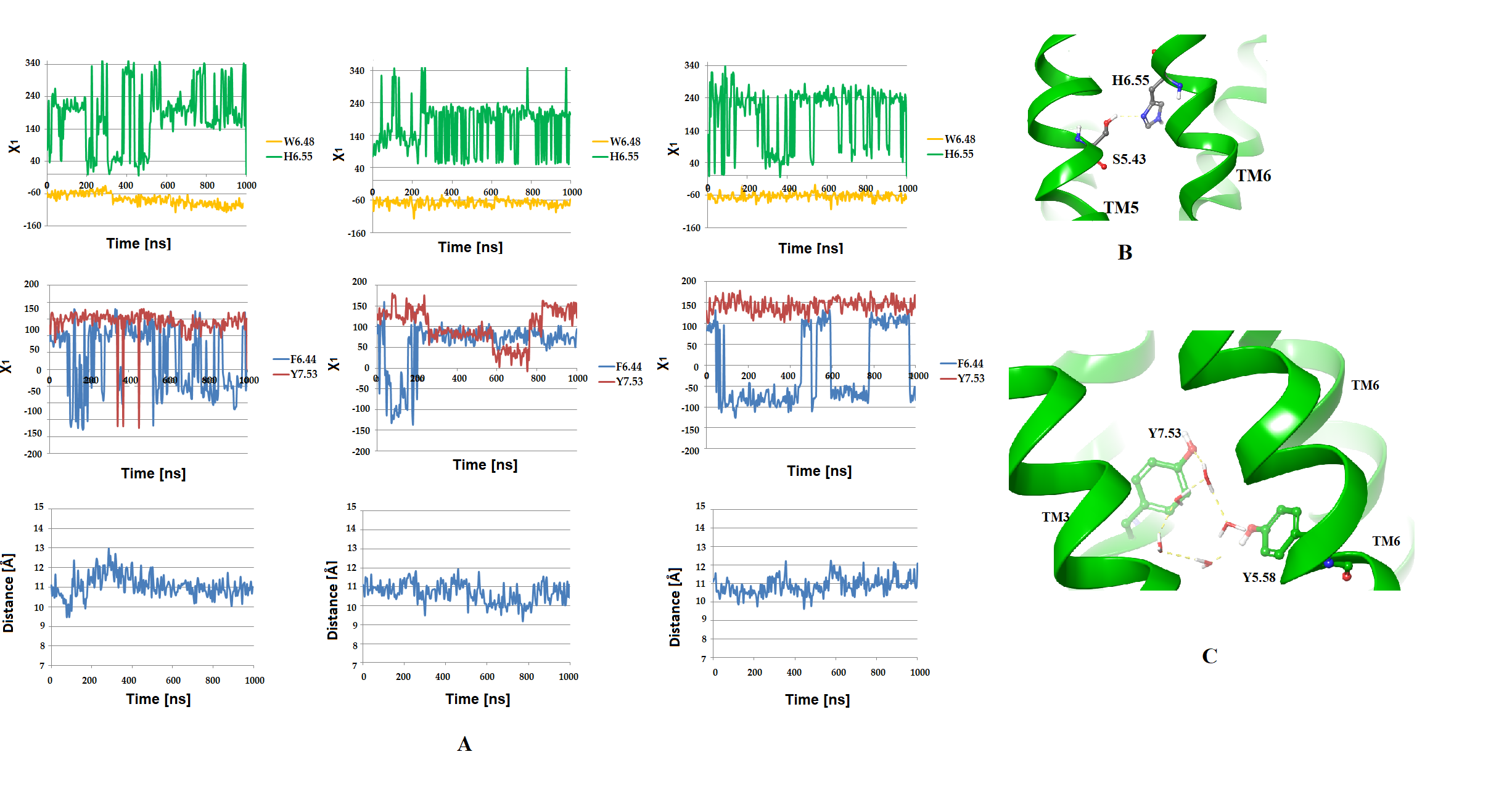


**Figure S7. A**: the side chain torsion angle changes of W6.48, H6.55, F6.44 and Y7.53 and distance of Y5.58 and Y7.53 for R2_DG2 complexes in three replicas during 1μs MD simulations. **B**: interaction of hydrogen bond (yellow dashes) between H6.55 and S5.43. Residues visualized as grey sticks with blue nitrogen and red oxygen atoms. **C**: the side chains of Y7.53 in relation to Y5.58. A (water-mediated) hydrogen bond showed as yellow dashes.


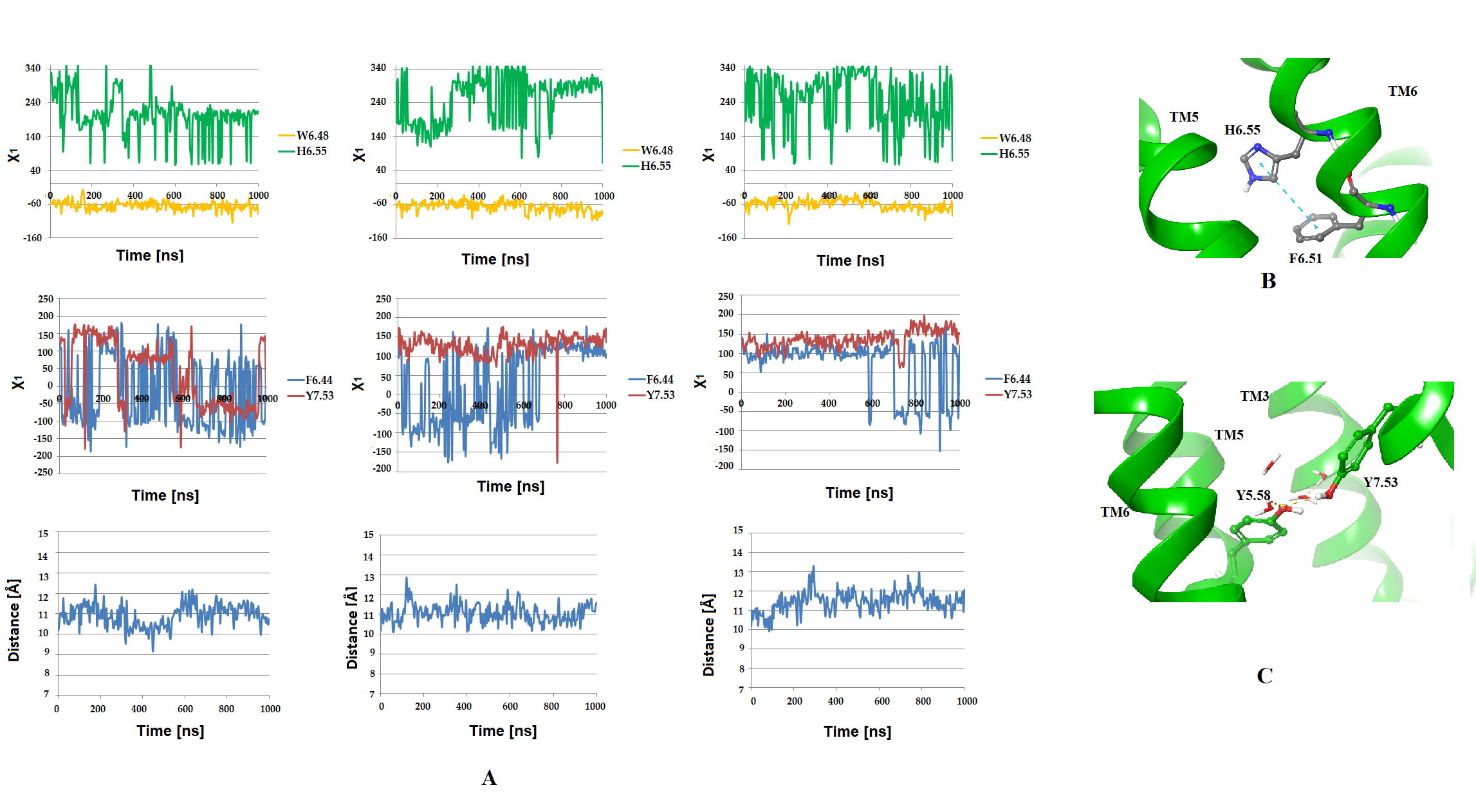


**Figure S8. A**: the side chain torsion angle changes of W6.48, H6.55, F6.44 and Y7.53 and distance of Y5.58 and Y7.53 for S2_DG2 complexes in three replicas during 1μs MD simulations. **B**: π -π interaction (blue dashes) between H6.55 and F6.51. Residues visualized as grey sticks with blue nitrogen and red oxygen atoms. **C**: the side chains of Y7.53 in relation to Y5.58. A (water-mediated) hydrogen bonds showed as yellow dashes.


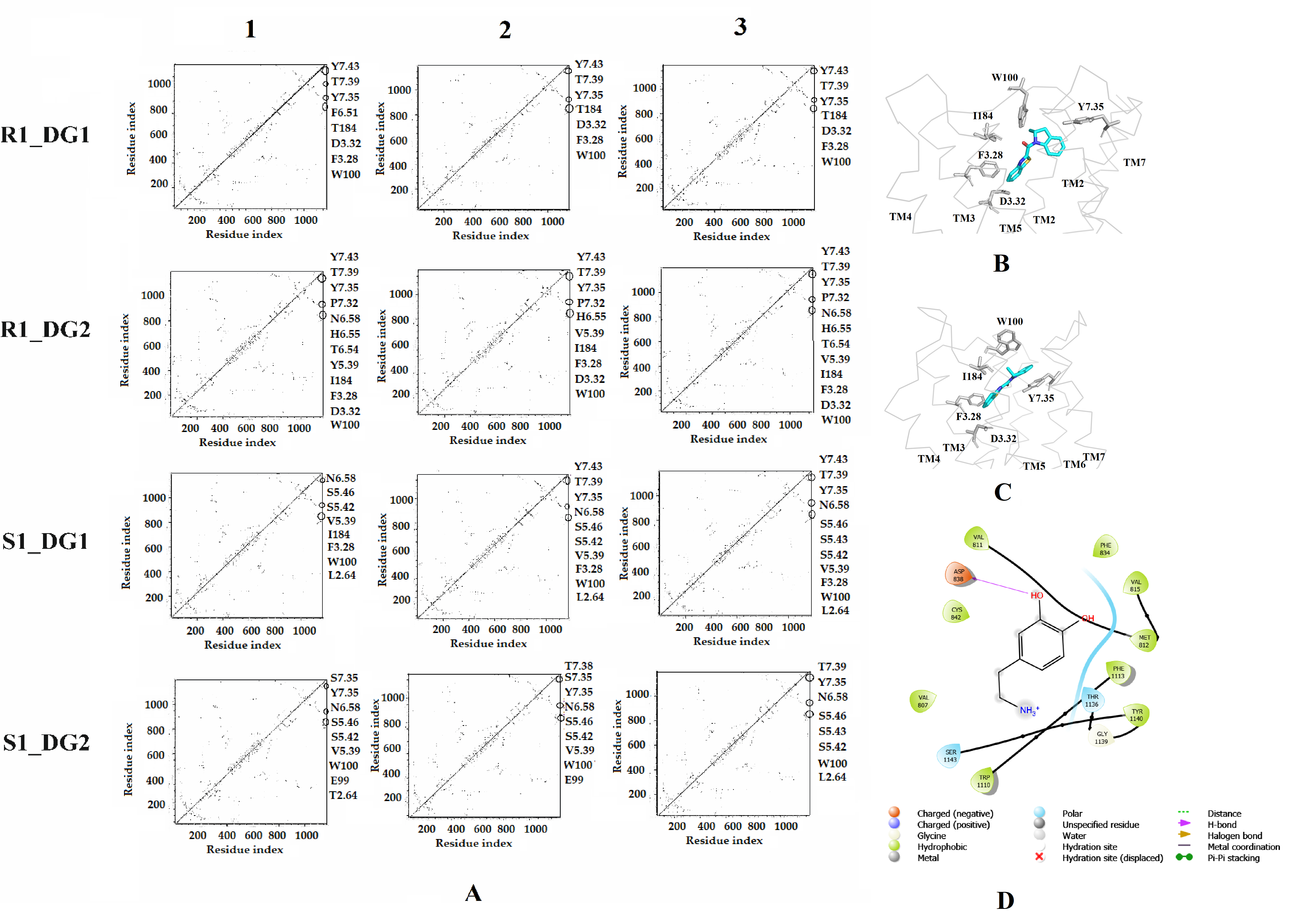


**Figure S9. A:** Distance matrices consisting of the distances between residue and ligand for the simulations with R1 and S1. The black dots correspond to points the length from 0–5 Å. The distance between modulators and D2 receptor residues are surrounded by a circle. **B:** the position of R1 in complex with DG1 (cyan stick representation) surrounded by crucial residues marked as a grey sticks. The helical bundle marked as a grey ribbon. **C:** the position of R1 in complex with DG2 (cyan stick representation) surrounded by crucial residues marked as a grey sticks. The helical bundle marked as a grey ribbon. **D**: Position of dopamine after 1µs MD simulations in S1_DG2 complex. The interactions shown on figure included in the corresponding legend.


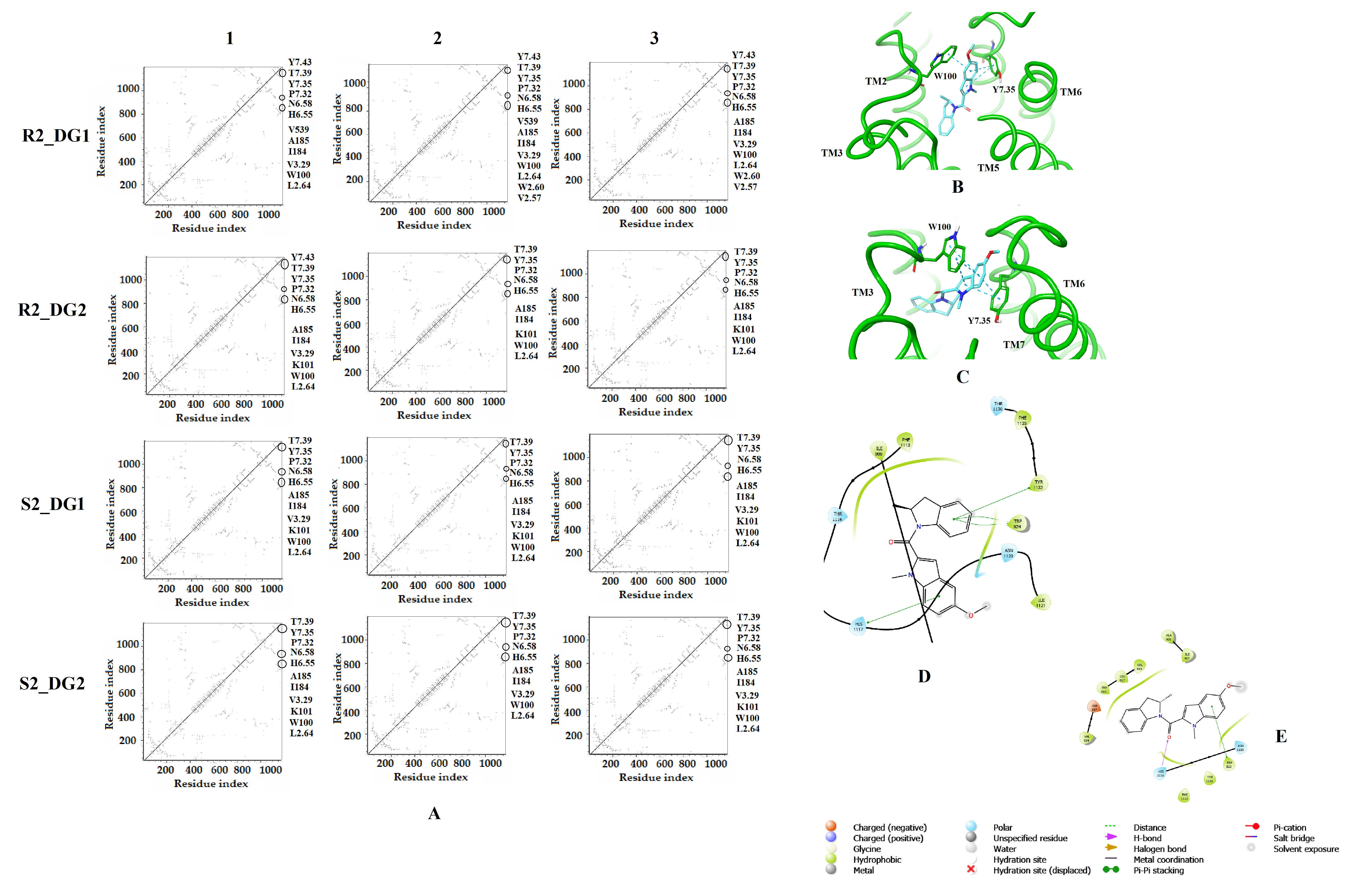


**Figure S10. A:** Distance matrices consisting of the distances between residue and ligand for the simulations with R1 and S1. The black dots correspond to points the length from 0–5 Å. The distance between modulators and D2 receptor residues are surrounded by a circle. **B:** the position of R2 in complex with DG1 (cyan stick representation) surrounded by crucial residues marked as a green sticks. The helical bundle marked as a green ribbon; π interactions are marked as a blue dashes. **C:** the position of R2 in complex with DG2 (cyan stick representation) surrounded by crucial residues marked as a green sticks. The helical bundle marked as a green ribbon; π interactions are marked as a blue dashes. **D**: the position of 2 in complex with DG1 surrounded by crucial residues. The interactions shown on figure included in the corresponding legend. **E**: the position of **2** in complex with DG1 surrounded by crucial residues. The interactions shown on figure included in the corresponding legend.


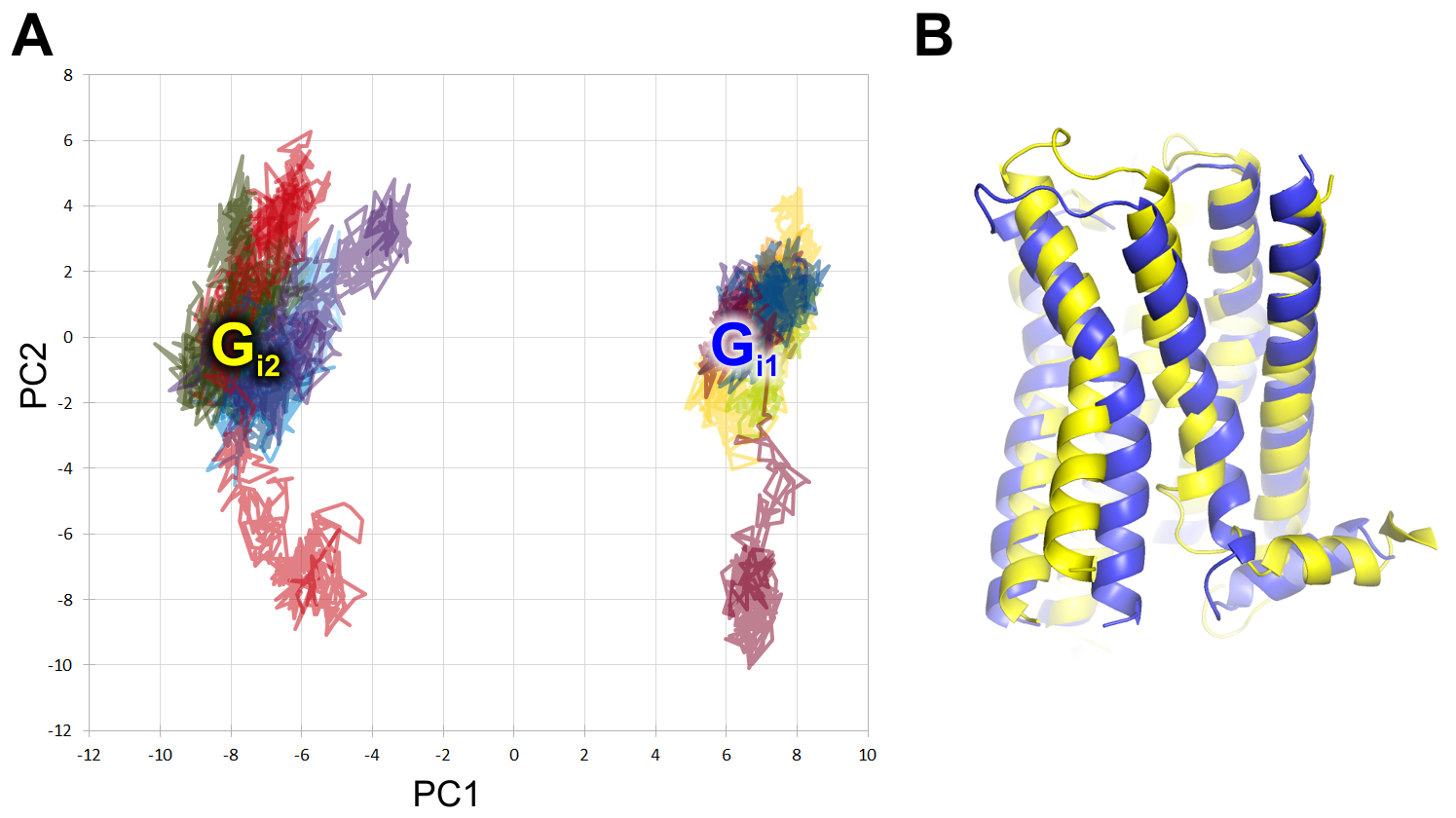


**Figure S11.** Principal component analysis calculated on all trajectories concatenated into one common space. **A**. Conformational space explored by G_i1_-containing complexes (on the right) and G_i2_-containing complexes (left). **B**. Superposition of extreme projections of PC1 on trajectories. Color coding corresponds to labels in panel A.

**Supplementary *in vitro* Methods**

**MTT assay.** The MTT (3-[4,5-dimethylthiazole-2-yl]-2,5-diphenyltetrazolium bromide) assay was performed on the mouse hippocampal neuronal cell line (HT-22; Merck, CA; Cat# SCC129; RRID: CVCL_0321), cultured in the medium recommended by the manufacturer (DMEM, Corning, USA) with L-glutamine, 4.5g/L glucose and sodium pyruvate.For the MTT assay, compound **2** was dissolved in DMSO to a 10mM stock solution from which dilutions were made in complete culture medium DMEM. The final DMSO percentage was equalized in each sample tested and was 0.4%, at the same time showing no toxicity to cells. Cells were seeded in a 96-well plate at a density of 3.0 × 10^3^ cells/cm^2^. After approximately 24h, cells were treated with pre-prepared concentrations of compound **2** ranging from 1-40 µM for 48h. After the incubation an MTT solution was added at a final concentration of 5 mg / ml. Then, a 4h incubation at 37 °C was performed and the absorbance at 590 nm (and 620 nm as a reference) was measured using a BioTek - Synergy ™ H1 microplate reader. Statistical analysis was performed using GraphPad Prism v. 7.0. The obtained results were presented as mean ± standard deviation. Differences between the control and study groups were assessed using one-way ANOVA of variance with Dunnett's post hoc test. The results considered statistically significant (P <0.05) are presented as: * - p <0.05; **- p <0.01; ***- p <0.001.

**Radioligand binding kinetic assays at D_2_ receptors.** A possible effect of compound **2** on the dissociation kinetics of the orthosteric radioligand [^3^H]-Spiperone was investigated in radioligand binding kinetic assays at D_2_ receptors. Assays were carried out in assay buffer (50 mM Tris-HCl, 120 mM NaCl, 5 mM KCl, 5 mM MgCl_2_, 1 mM EDTA, pH = 7.4) on membranes from CHO-K1 cells stably expressing the cloned human D_2S_ receptor and using 0.2 nM [^3^H]-Spiperone. Radioligand was allowed to equilibrate with the membranes for 2 hours at 30ºC in assay buffer prior to the addition of 10 µM sulpiride as dissociator. Compound **2** (10 µM) was preincubated with the membranes for 1 hour and it continued present during the equilibration of the radioligand binding prior to the addition of the dissociator. Vehicle (0.1% DMSO) was included as control in the experiments. Dissociation was carried out at different time points for 180 min. Nonspecific binding was assessed in the presence of 10 µM sulpiride. Reactions were terminated by rapid filtration through glass fiber filters GF/C presoaked in a 0.5% (w/v) poly(ethyleneimine) solution, the filters were washed 4 times with ice-cold wash buffer (50 mM Tris-HCl, 0.9% NaCl, pH = 7.4), and the radioactivity was quantified in a MicroBeta² microplate liquid scintillation counter (Perkin Elmer, Waltham, MA, USA).

**Supplementary *in vitro* Results**


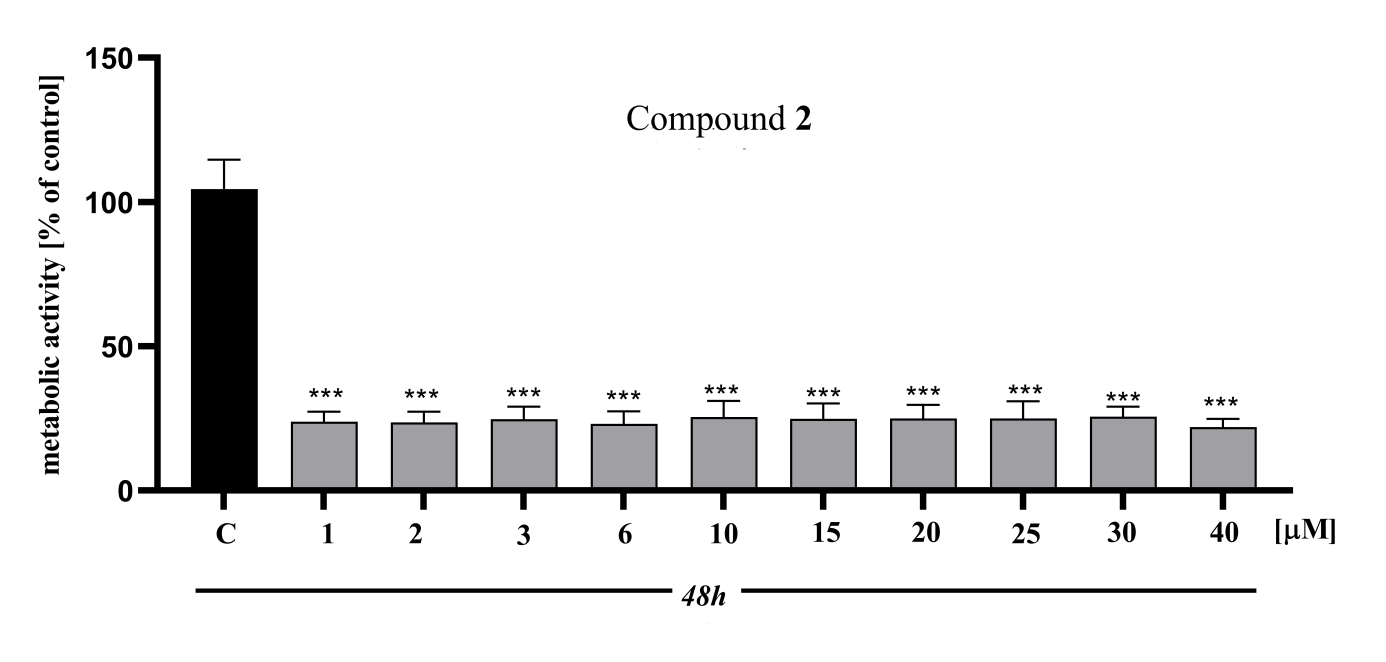


**Figure S12.** The cytotoxic activity of compound **2** on the metabolic activity of mouse hippocampal neurons (cell line HT-22) as measured in the MTT assay. In the entire concentration range (1-40 µM) compound **2** showed toxicity towards neurons by the significant reduction of their viability.


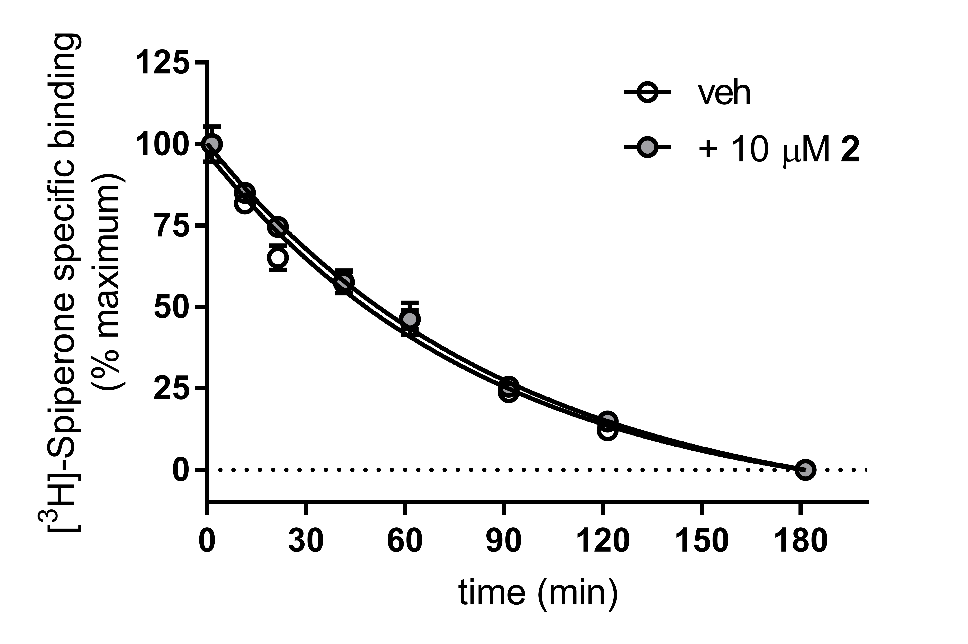


**Figure S13.** Effect of compound **2** on dissociation kinetics of [^3^H]-Spiperone: compound **2** did not altered the dissociation rate constant (*k*_off_) of the radioligand. Vehicle (veh, 0.1% DMSO) or 10 µM compound **2** were preincubated for 1 hour and continued present during the equilibration of the radioligand binding prior to the addition of the dissociator. The graph shows the data (mean ± SEM) of one experiment performed in duplicate. Dissociation rate constant (*k*_off_) of [^3^H]-Spiperone in these assays was (mean ± SEM) 0.012 ± 0.002 min^-1^ and 0.011 ± 0.001 min^-1^ for control vehicle and compound **2**, respectively.


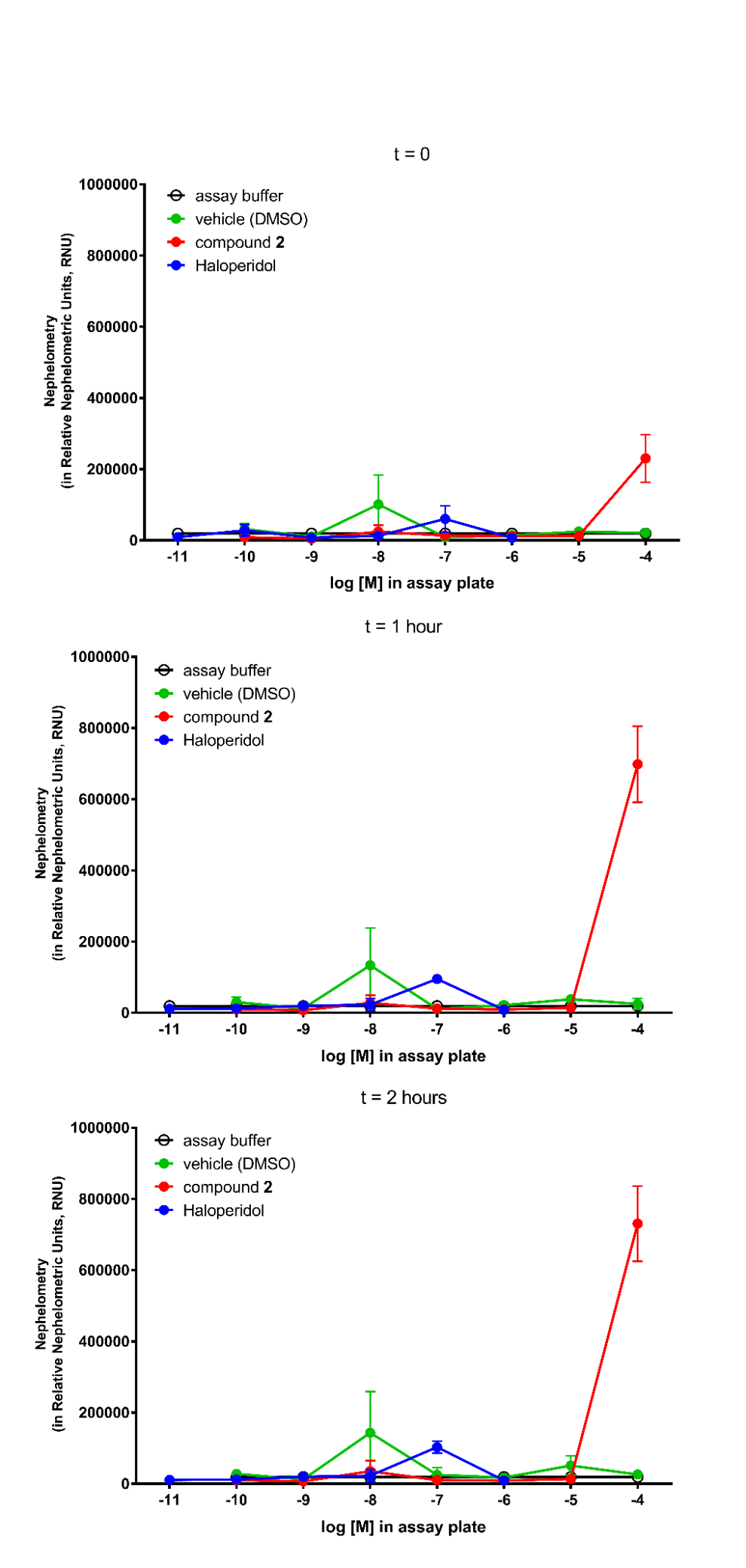


**Figure S14**. Solubility of compound **2** in radioligand binding assays in assay plate conditions.


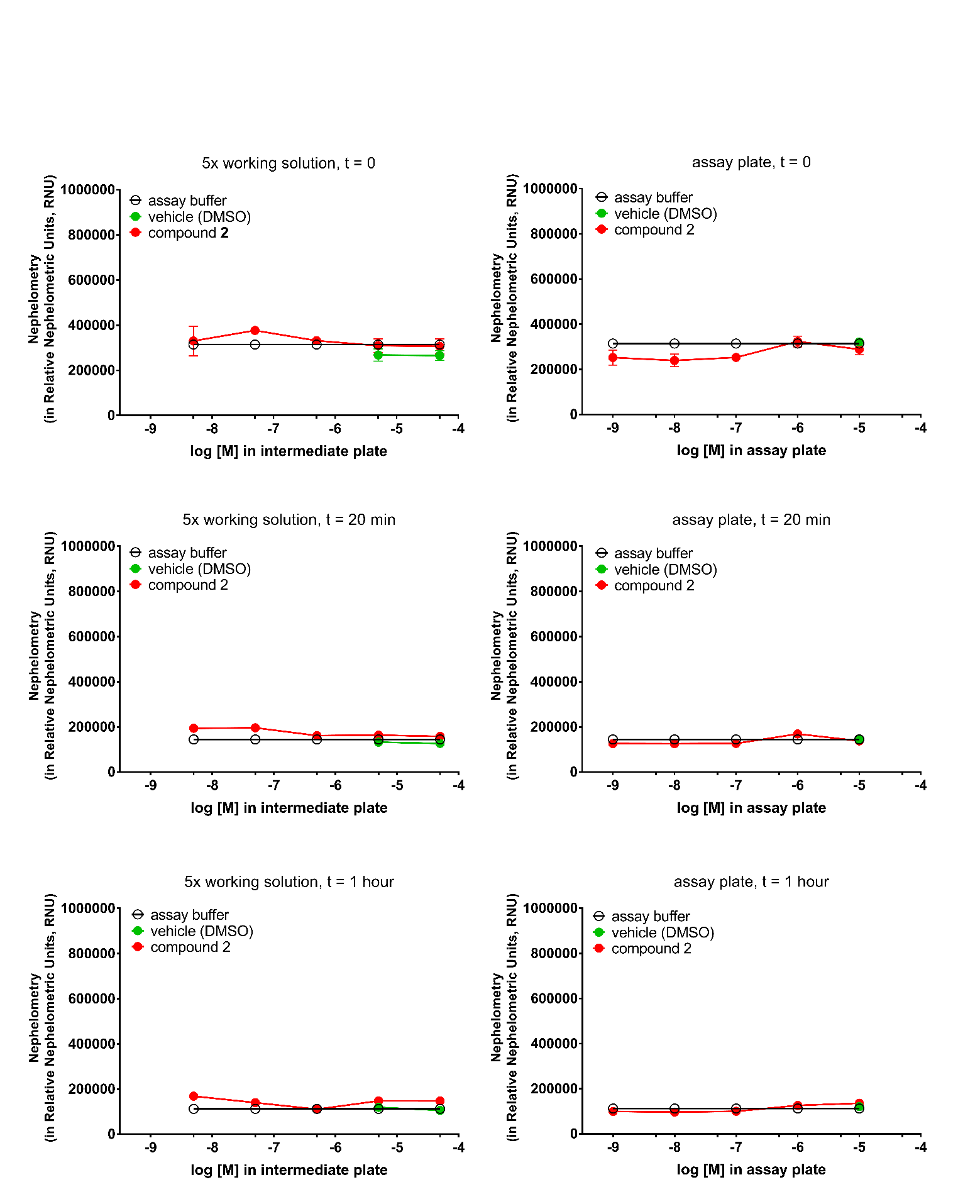


**Figure S15**. Solubility of compound **2** in cAMP assays, either in the 5x intermediate working solution or in the assay.

**Table S1.** Similarity of compound **1** and **2** to the known dopamine D_2_ receptor ligands available in the CHEMBL database.

| Compound | CHEMBL ID of most similar compound | 2D structure of most similar compound | Similarity |
| --- | --- | --- | --- |
|  | CHEMBL320597 |  | 0.247 |
|  | CHEMBL106916 |  | 0.247 |
|  | CHEMBL104058 |  | 0.247 |
|  | CHEMBL319043 |  | 0.250 |

**Table S2.** Possible pharmacological properties of compounds **1** and **2** predicted by PASS software.

| Compound | Pharmacological Effects | Mechanisms of Action | Anti-targets | Toxic and adverse effects |
| --- | --- | --- | --- | --- |
| **1** | Nootropic (0.573)  Antiparkinsonian (0.469)  Alzheimer's disease treatment (0.456)  Attention deficit (0.428)  Dementia treatment (0.420)  Vascular dementia treatment (0.417) | Nootropic (0.573)  MAP kinase 10 inhibitor (0.488) | No anti-targets with probability above 0.4 found | No toxic or adverse effects with probability above 0.4 found |
| **2** | No effects with probability above 0.4 found | 5-Hydroxytryptamine release stimulant (0.523) | No anti-targets with probability above 0.4 found | No toxic or adverse effects with probability above 0.4 found |

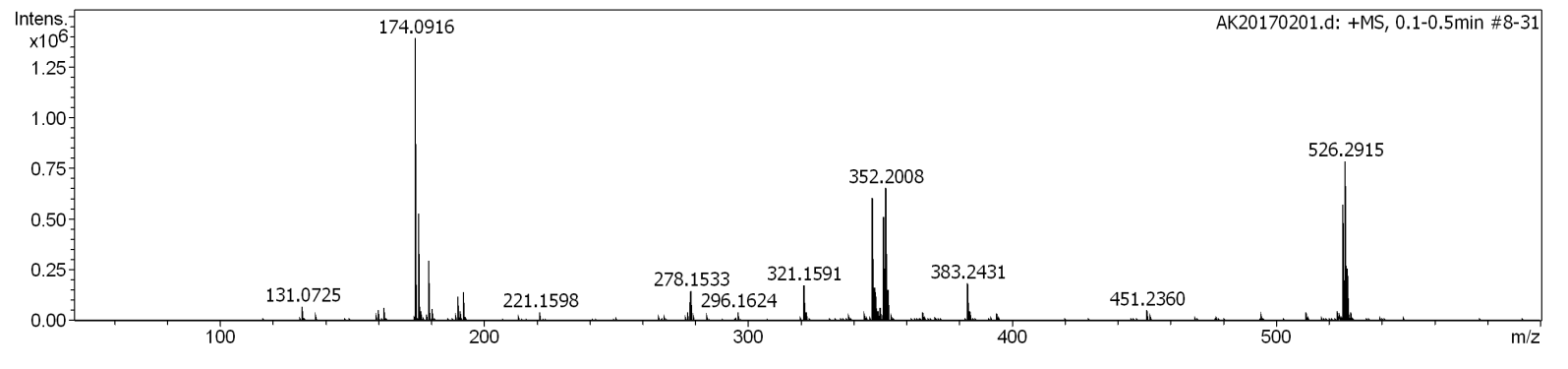

Supplement: Supplementary file 1 — Supplementary file1 (DOCX 8788 KB) [file 43440_2021_352_MOESM1_ESM.docx]
